# Ultrahigh-throughput screening-assisted in vivo directed evolution for enzyme engineering

**DOI:** 10.1186/s13068-024-02457-w

**Published:** 2024-01-22

**Authors:** Shuaili Chen, Zhanhao Yang, Ze Zhong, Shiqin Yu, Jingwen Zhou, Jianghua Li, Guocheng Du, Guoqiang Zhang

**Affiliations:** 1 Science Center for Future Foods, School of Biotechnology, Jiangnan University, 1800 Lihu Road, Wuxi, 214122 Jiangsu China; 2 National Engineering Research Center for Cereal Fermentation and Food Biomanufacturing, Jiangnan University, 1800 Lihu Road, Wuxi, 214122 Jiangsu China; 3 Engineering Research Center of Ministry of Education On Food Synthetic Biotechnology, Jiangnan University, 1800 Lihu Road, Wuxi, 214122 Jiangsu China; 4 Jiangsu Province Engineering Research Center of Food Synthetic Biotechnology, Jiangnan University, 1800 Lihu Road, Wuxi, 214122 Jiangsu China

**Keywords:** Thermosensitive regulator, Targeted mutagenesis, In vivo directed evolution, Biosensor, Ultrahigh-throughput screening

## Abstract

**Background:**

Classical directed evolution is a powerful approach for engineering biomolecules with improved or novel functions. However, it traditionally relies on labour- and time-intensive iterative cycles, due in part to the need for multiple molecular biology steps, including DNA transformation, and limited screening throughput.

**Results:**

In this study, we present an ultrahigh throughput in vivo continuous directed evolution system with thermosensitive inducible tunability, which is based on error-prone DNA polymerase expression modulated by engineered thermal-responsive repressor cI857, and genomic MutS mutant with temperature-sensitive defect for fixation of mutations in *Escherichia coli*. We demonstrated the success of the in vivo evolution platform with β-lactamase as a model, with an approximately 600-fold increase in the targeted mutation rate. Furthermore, the platform was combined with ultrahigh-throughput screening methods and employed to evolve α-amylase and the resveratrol biosynthetic pathway. After iterative rounds of enrichment, a mutant with a 48.3% improvement in α-amylase activity was identified via microfluidic droplet screening. In addition, when coupled with an in vivo biosensor in the resveratrol biosynthetic pathway, a variant with 1.7-fold higher resveratrol production was selected by fluorescence-activated cell sorting.

**Conclusions:**

In this study, thermal-responsive targeted mutagenesis coupled with ultrahigh-throughput screening was developed for the rapid evolution of enzymes and biosynthetic pathways.

**Supplementary Information:**

The online version contains supplementary material available at 10.1186/s13068-024-02457-w.

## Background

With the development of enzyme engineering and synthetic biology technology, renewable raw materials can be converted into value-added chemicals using biocatalysis or microbial cell factories [1–3]. However, due to insufficient enzyme stability, catalytic efficiency and selectivity, the requirements of industrial biocatalysis cannot be met. Directed evolution, which has been successfully developed in recent decades, is a powerful tool to guide protein evolution and improve the synthesis efficiency of metabolic pathways as well as the performance of microbial cell factories [4, 5]. However, classical directed evolution methods rely on manual operation for rounds of stepwise processes, including DNA extraction, in vitro mutagenesis, transformation and selection, which are usually labour intensive and limit the efficiency, scale and depth of evolution [5–7].

In recent years, in vivo continuous evolution has attracted increasing attention. This process simulates the lengthy process of natural evolution in the laboratory with rapid in vivo diversification and autonomous functional selection, thus permitting exploration of a broader sequence space with reduced human intervention and effort [6, 8, 9]. Various in vivo mutagenic systems have been exploited, from viruses to bacteria and eukaryotes. Phage-assisted continuous evolution (PACE) is a general system for evolving proteins coupled with pIII protein expression in *E. coli* that relies on viral replication and chemostat culture [10–12]. A plasmid mutagenesis system mediated by low-fidelity DNA repair polymerase I (Pol I) was established in *Escherichia coli* and was applied to generate TEM-1 β-lactamase mutants conferring aztreonam resistance [13–15]. Recently, a highly orthogonal error-prone DNAP-plasmid pair (OrthoRep) was developed for restricting mutagenesis to a linear cytoplasmic plasmid without interfering in genome replication in *Saccharomyces cerevisiae* [16, 17]. OrthoRep has been applied to engineer a significant number of proteins, including dihydrofolate reductase [17], tryptophan synthase β-subunit [18] and sulfide-dependent thiazole synthase [19]. In these in vivo continuous evolution systems, user-defined genes are usually mutated by inducing the expression of mutator genes, which is conducive to reducing unexpected mutations and enabling the spatiotemporal regulation of increased mutagenesis rates. In most cases, in vivo mutagenesis tools were used to couple the activity of the evolved protein with cell growth or survival, allowing the automatic and direct elimination of undesired variants under selective pressure. However, protein activity is typically a non-selectable trait, especially for industrial enzymes and metabolic pathway enzymes.

In recent years, numerous transcription factor (TF)-based biosensors have been exploited as reporters to regulate fluorescent protein expression according to changes in metabolite concentration, offering an ultrahigh-throughput screening method based on fluorescence-activated cell sorting (FACS) [20, 21]. Droplet-based microfluidic technology provides an attractive alternative for signal detection related to secretory protein activity by encapsulating single cells in spatially segregated droplets [22–25]. Thus, the fluorescence signal generated by the substrate or product and biosensors links genotype and phenotype by combining enzyme activity directly or indirectly with ultrahigh-throughput screening to obtain the desired performance in a cost-effective and efficient way. In short, ultrahigh-throughput continuous evolution systems enable rapid dramatic improvements in enzymatic activity or pathway flux from large variant libraries.

In this study, an in vivo continuous evolution system was developed based on DNA Pol I and a temperature-sensitive variant of the mismatch repair protein MutS in *E. coli*. Temperature upshift, permitting the expression of error-prone DNA Pol I and temporary defects in mismatch repair machinery, enabled increased plasmid mutagenesis. The in vivo mutagenesis system was then combined with ultrahigh-throughput screening and applied to the rapid evolution of α-amylase and resveratrol biosynthesis pathway. Significant improvements in α-amylase activity and resveratrol titer were acquired in a short time, thereby demonstrating the accelerated evolution of specific phenotypes.

## Results

### Directed evolution of the highly thermosensitive repressor cI857

A dynamic regulatory element with rapid responsiveness and scalable control activity is required for a regulatable in vivo evolution system. The thermo-regulated expression system λP_R_/P_L_–cI857 has been widely used to produce heterologous proteins in bacteria [26]. cI857 is a temperature-sensitive mutant (cI A67T) of the cI repressor protein from λ phage. cI857 forms dimers that can bind to the P_R_/P_L_ promoter region at a lower temperature, such as 28–30 °C, retaining its wild-type function and blocking the transcription of downstream genes. At temperatures of 37 °C or above, cI857 dimers dissociate from the promoter region, which permits RNA polymerase to bind to the promoter region and initiate gene transcription, especially at 40–42 °C, which is the more efficient temperature for cI857 dimer inactivation [26, 27].

**Fig. 1:**
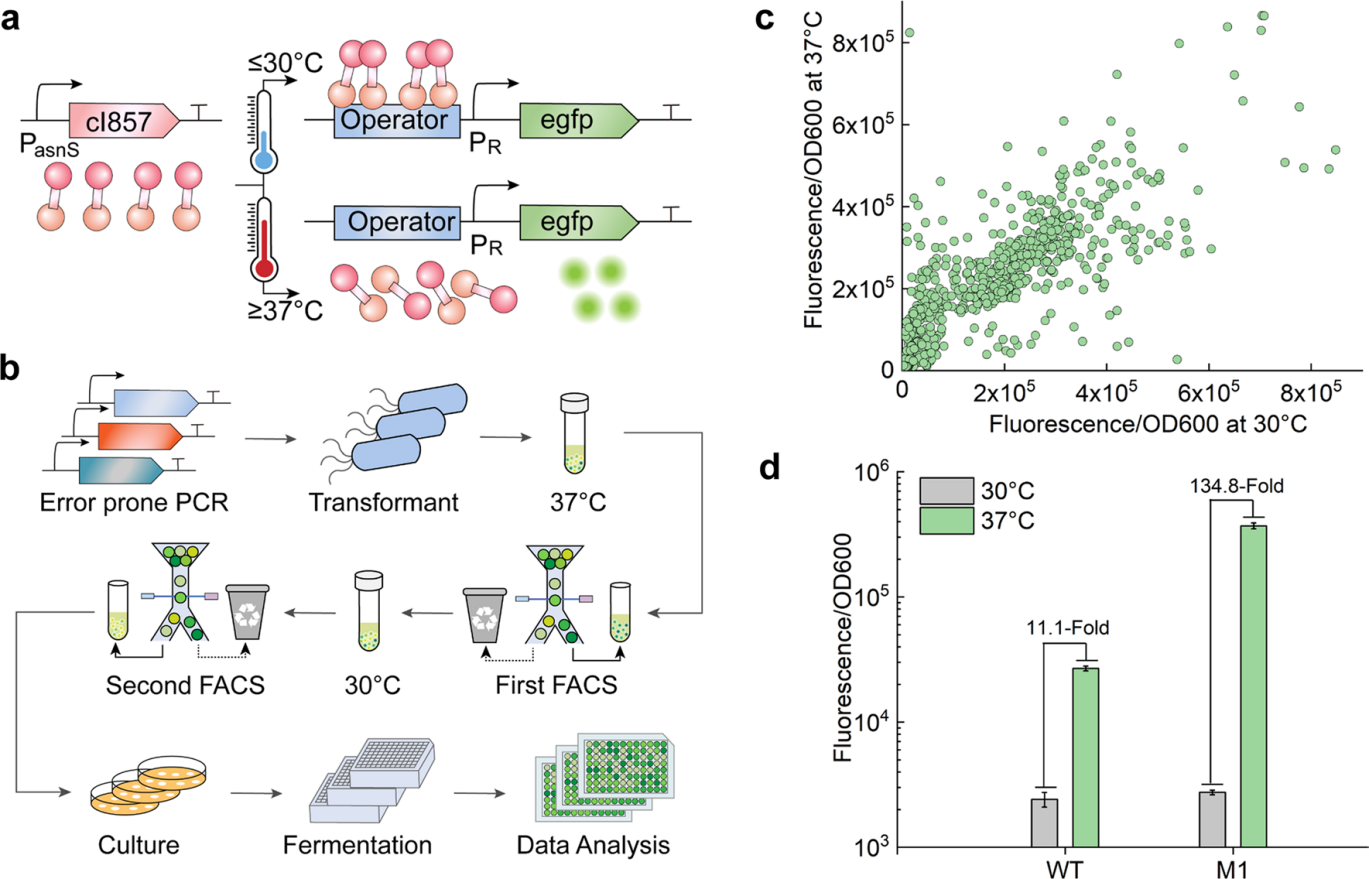
Directed evolution for thermosensitive repressor cI857. **a** Working principle of λP_R_-cI857 system. In the expression cassette, the repressor gene cI857 was constitutively expressed. The inducible promoter P_R_ controlled the expression of EGFP. **b** General schematic procedure for the directed evolution of cI857. **c** Fluorescence intensity of the sorted 768 cells after fermentation for 6 h in 96 deep well plate. **d** Fluorescence intensity of mutant M1 and wild-type strain after 9 h of fermentation in the shake flask at 30 ℃/37 ℃

To achieve the efficient switch ON/OFF for mutator Pol I, λPR–cI857 system with the lower leakage at 30 ℃ and higher expression strength at 37 ℃ is preferable. Thus, directed evolution of cI857 was first carried out, and higher temperature-sensitive and low-leakage mutants were selected through fluorescence screening (Fig. 1a). A library of cI857 mutant genes was constructed downstream of the constitutive promoter–5′-UTR complex (PUTR) asnS [28], with enhanced green fluorescent protein (EGFP) expression controlled by the P_R_ promoter. After culturing the library of cells carrying cI857 mutants at 37 °C, at least 10,000 events with higher fluorescence, indicating stronger induction of the thermal-responsive switch, were collected in liquid LB medium through the first round of FACS. To reduce expression leakage at the repressive temperature, the second round of FACS was performed, and approximately 5,000 events showing lower fluorescence were sorted from the collected cells cultured at 30 °C (Fig. 1b). Finally, 768 strains were selected for the fluorescence assay, with the wild-type strain used as the control strain (Fig. 1c). An evolved mutant (M1) with lower fluorescence at 30 °C and higher fluorescence at 37 °C was isolated for plasmid extraction. Compared with wild-type cI857, the cI857 mutant (cI857*) from the M1 strain has three changes in the DNA sequence, including two substitutions and one deletion (ΔT57, A400T and T418A). The repression effect of the M1 strain was basically the same as that of the wild-type strain (WT) at 30 °C, while the induction effect at 37 °C was greatly improved at 3 h, 6 h and 9 h (Fig. 1d, Additional file 1: Fig. S1a and b). At 9 h, the fluorescent protein expression of the M1 strain at 37 ℃ was 134.8 times that at 30 ℃, and the induction effect was approximately 13.8 times that of the wild-type strain. The results suggested that cI857* provided significantly increased protein expression at the same induction temperature and could be used as a more efficient temperature-controlled heterologous protein expression system.

### Temperature-controlled in vivo evolution based on DNA Pol I in *E. coli*

**Fig. 2:**
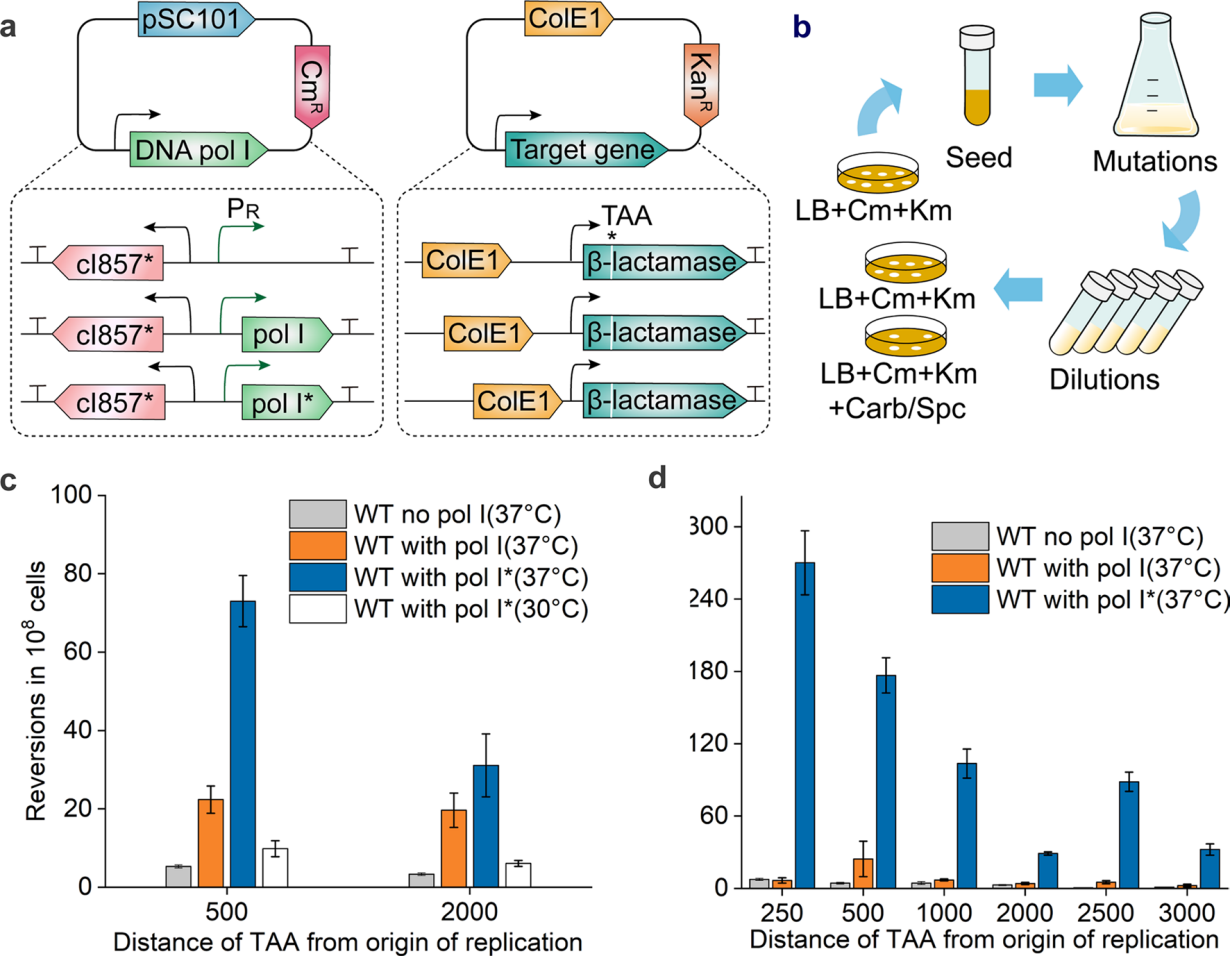
Construction and characterization of temperature-controlled in vivo evolution system in *E. coli.* **a** Construction of the two plasmids for in vivo mutagenesis. The expression of DNA Pol I* was controlled by a thermal-responsive P_R_ promoter. **b** Reversion tests were implemented after the two plasmids were cotransformed into *E. coli* BL21 (DE3). Cm, chloramphenicol; Km, kanamycin; Carb, carbenicillin; Spc, spectinomycin. **c** Carbenicillin-resistance reversion rate of wild-type strain with/without pol I or pol I* gene. **d **Reversion rate of cells from the saturated culture at different distances from ColE1 ori

Previously, a highly mutagenic DNA polymerase I (Pol I D424A I709N A759R, Pol I*) was reported to erroneously replicate a region located downstream of the origin of replication (ori), relying on the Pol I specifically required for ColE1 plasmid replication in *E. coli* [15]. To develop a temperature-controlled in vivo evolution system, a two-plasmid system was constructed based on Pol I* in *E. coli* BL21 (DE3) (Fig. 2a). The first plasmid is the low-copy mutator plasmid pSC101, the replication of which does not depend on Pol I, carrying the mutator gene pol I* under the control of a thermal-responsive P_R_ promoter. The second plasmid is the multicopy target plasmid pET28a containing ColE1 ori and downstream genes of interest, that is, genes to be mutated. To sensitively assess the occurrence of mutations, a premature nonsense codon (TAA) was introduced into a constitutively expressed β-lactamase gene, leading to early termination of translation and consequently a carbenicillin-sensitive phenotype. Plasmid pTA500 was constructed to locate the premature TAA codon of the expression cassette at 500 bp downstream of ColE1 ori, allowing cells to reacquire antibiotic resistance when mutations arise to generate permissive codons at the TAA. Therefore, the targeted mutation rate could be quantified by the frequency of carbenicillin-resistant colonies (Fig. 2b).

To ensure that the P_R_-cI857 system achieved good temperature response in low copy plasmids, cI857 or cI857* was constitutively expressed from plasmid pSC101 under the control of a higher strength PUTR consisting of cascade PUTR, PUTR_ssrA_–PUTR_infC‑rplT_ [28], resulting in plasmid pSC101–cI857(*)–λPR–EGFP (Additional file 1: Fig. S1c). The fluorescence assay showed that the strain bearing cI857* presented a more dramatic decrease in expression leakage at 30 °C and a modest but unmistakable increase in inducible gene expression at 37 °C compared to the strain harboring cI857, consistent with the results indicated above (Additional file 1: Fig. S1d). Accordingly, the P_R_–cI857* thermal regulation system was more suitable for the regulation of Pol I* expression and repression. The resulting plasmid Pol I* was cotransformed into *E. coli* BL21 (DE3) with the target plasmids pTA500 and pTA2000, respectively. The strain containing the Pol I* plasmid showed a significantly lower reversion rate at 30 ℃ than at the induction condition of 37 ℃, and the rate at 30 °C was comparable to that of the control strain without the Pol I gene, exhibiting tightly regulated Pol I* expression (Fig. 2c). Simultaneously, another constitutive expression cassette encoding the spectinomycin resistance gene *aadA* with the premature stop codon TAA was assembled into the target plasmid to generate plasmids pTS250 and pTS1000 for the reversion assay. The general similarity between carbenicillin and spectinomycin resistance reversion frequency suggested that the two-plasmid system was an effective and temperature-controlled mutagenesis system (Additional file 1: Fig. S2a).

As reported previously, whether cultures were grown to saturation was also a key factor influencing mutagenesis [15]. Considering that ColE1 plasmid replication is involved in partial regions of the entire plasmid due to the gradual switch between Pol I and Pol III in *E. coli* [13, 29], the length of the region susceptible to mutagenesis by Pol I* was assessed by the reversion of fresh saturated cultures bearing a pTA plasmid with the premature TAA codon at different distances downstream of ColE1 ori. Notably, the wild-type strain with the Pol I* plasmid exhibited the highest reversion rate at 250 bp from ColE1 ori, approximately 41 times that of the strain with the Pol I plasmid, and effectively gave rise to mutations within at least 1 kb despite the sharply decreased reversion frequency with increasing distance from ori (Fig. 2d, Additional file 1: Fig. S2b). The results indicated that the distance from ori impacted the mutation rate, in agreement with previous reports [15, 29, 30].

### Synergistic action with MutS to accelerate the evolution rate

**Fig. 3:**
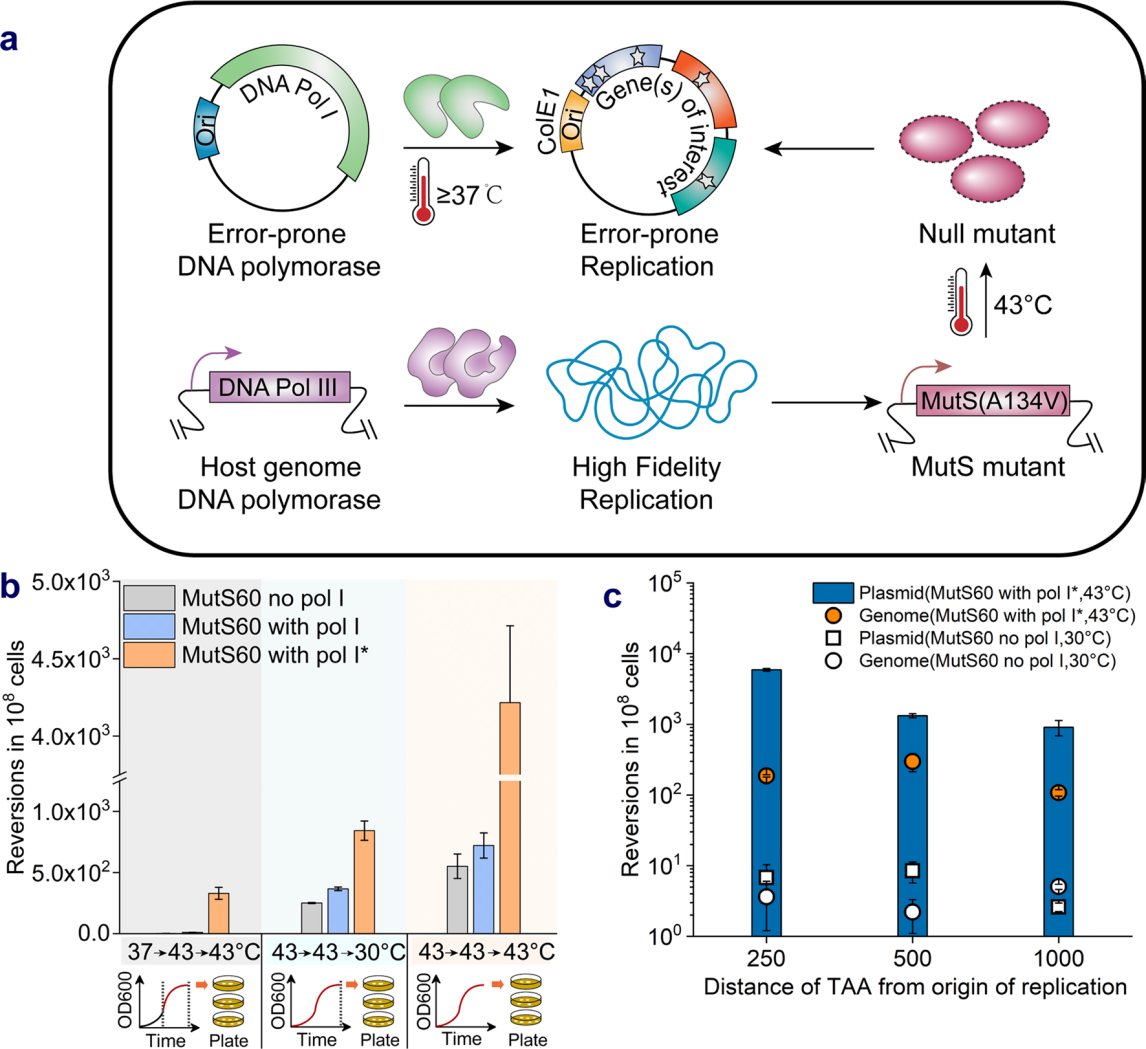
Modification of genomic *mut*S to increase plasmid mutation rate. **a** Error-prone replication of target plasmid was influenced by synergistic action of the expression of DNA Pol I* and MutS mutant induced by temperature shift. **b** Reversion rate of MutS60 strain carrying pol I or pol I* gene at different temperatures for mutagenesis. For mutagenesis of the first experimental group, the cultures were grown for 10 h until at mid-exponential phase at 37 ℃, cultured to saturation for another 8 h at 43 ℃, and incubated on LB plates at 43 ℃. For the second group, the cultures were grown to saturation for 20 h at 43 ℃ and then incubated on LB plates at 30 ℃. For the third group, the temperature for both shake flask and plate growth was remained at 43 ℃. **c** The reversion rate of MutS60 strain containing pol I* gene at different distances from ColE1 ori

The DNA mismatch repair (MMR) system is dedicated to checking errors in newly replicated DNA strands, maintaining a relatively low mutation rate in the host genome. In the mismatch repair system of *E. coli*, MutS recognizes the base mismatch, and MutH, recruited by MutL, cleaves the corresponding strand after interacting with MutS [31]. Overexpressing dominant-negative allele *mutL* E32K or knocking out one or more of these proteins in *E. coli* has been used to improve the mutation rate and mutations fixation [13, 32, 33]. To avoid the accumulation of deleterious mutations in host genome, precise spatiotemporal control of enhanced mutagenesis must be considered. Here, to further elevate the mutation rate in the target plasmid, the MMR system was modified by introducing the *mutS* A134V mutation into BL21 (DE3) to acquire a temperature-sensitive MutS mutant, MutS60 [34]. MutS60 exhibited almost wild-type levels of spontaneous mutagenesis at 37 °C, but dramatic increase in the mutation rate at 43 °C [34]. Pol I* and pTA2000 plasmids were cotransformed into the MutS60 strain (Fig. 3a). After culture at different temperatures, the mutation frequency of MutS60 with the Pol I* plasmid at 30 ℃ was lower than that at 37 ℃. Moreover, the combination of Pol I* and MutS60 at 43 °C resulted in a higher reversion rate (Additional file 1: Fig. S2c).

Because the culture temperature affects bacterial growth as well as the plasmid mutation rate, the effect of temperature on targeted mutagenesis was characterized. As shown in Fig. 3b, when the mutagenesis temperature maintained at 43 °C, MutS60 strain with Pol I* gene showed the optimal plasmid mutation rate. According to the optimized culture temperature, the mutation frequency of strain MutS60 with Pol I* was significantly improved at different distances from ori (Fig. 3c), approximately 638 times that of the wild-type strain with Pol I at 250 bp. Based on the assumption of even mutational density, the mutation rate of the target plasmid was determined to be 6.77 × 10^–8^ per nucleotide per cell per generation, ~ 600-fold higher than the background mutation rate in *E. coli*. In addition, a rifampicin-resistance reversion assay was used to evaluate the impact of the in vivo mutagenesis system on the genome, since mutations occurring in the RNA polymerase β subunit encoded by the genomic rpoB gene can relieve the inhibition of RNA polymerase activity by rifampicin, enabling transcription and cell survival [35]. The results indicated that the mutation rate of the *E. coli* genome was increased by about 70-fold during mutagenesis (Fig. 3c). Notably, plasmid extraction and retransformation procedures are necessary after mutagenesis, especially multiple rounds of mutagenesis to prevent off-target genomic mutagenesis from interfering with selection.

### Mutational spectrum analysis

To characterize the nucleotide changes in the target plasmid, the β-lactamase genes from mutants with recovered carbenicillin resistance were sequenced. Approximately 50 resistant reversion mutants at a distance of 250 bp from ori were randomly selected and from position 100 to 750 bp downstream of ori was sequenced. Of the 49 clones, 47 had mutations at the TAA stop codon. Among the substitution mutations, TTA (61%) was the predominant type, followed by TCA (18%) and CAA (14%) (Table 1). To further characterize the mutation spectrum, the colonies with spectinomycin resistance were sequenced. All of the 50 clones were TTA (100%) substitution mutations (Additional file 1: Table S1), whose encoded amino acid was Leu33, the same as that in the wild-type aadA. It is speculated that Leu33 interacts with nearby key amino acids and thus has an important impact on the conformation and enzyme activity of *aad*A, restricting base substitution diversity [36]. In conclusion, TAA → TTA is the main substitution related to Pol I*, consistent with previous studies [15]. After introducing MutS60, the mutational spectrum was changed, and TAA was mostly replaced by CAA (85%) (Table 2). This difference may be connected to the stronger preference for conversion in mismatch repair-deficient cells [37].

**Table 1:** Mutation spectrum at TAA of wild-type strain with Pol I* plasmid (β-lactamase as reporter)

Nucleotide changes	Amino acid changes	Count	Frequency (%)
TAA → TTA	STOP → L	30	61.2
TAA → TCA	STOP → S	9	18.4
TAA → CAA	STOP → Q	7	14.3
TAA → CTA	STOP → L	1	2
TAA → TAA	STOP → STOP	2	4
Total	–	49	100

**Table 2:** Mutation spectrum at TAA of MutS60 with Pol I* plasmid (aadA as reporter)

Nucleotide changes	Amino acid changes	Count	Frequency (%)
TAA → CAA	STOP → Q	41	85
TAA → AAA	STOP → K	5	11
TAA → TAC	STOP → Y	2	4
Total	–	48	100

### Microfluidic droplet screening-assisted in vivo evolution of α-amylase

α-Amylase is widely used in the food, brewing and pharmaceutical industries, leading to great market demand. However, the acid susceptibility, thermal instability and relatively low activity of α-amylase often make it difficult to meet the demands for industrial applications. Therefore, accelerating the directed evolution of α-amylase would be of great significance for the improvement of the amylase industry and even the development of biocatalysts. Here, the in vivo mutagenesis system was applied in combination with droplet microfluidic screening to enhance α-amylase activity.

**Fig. 4:**
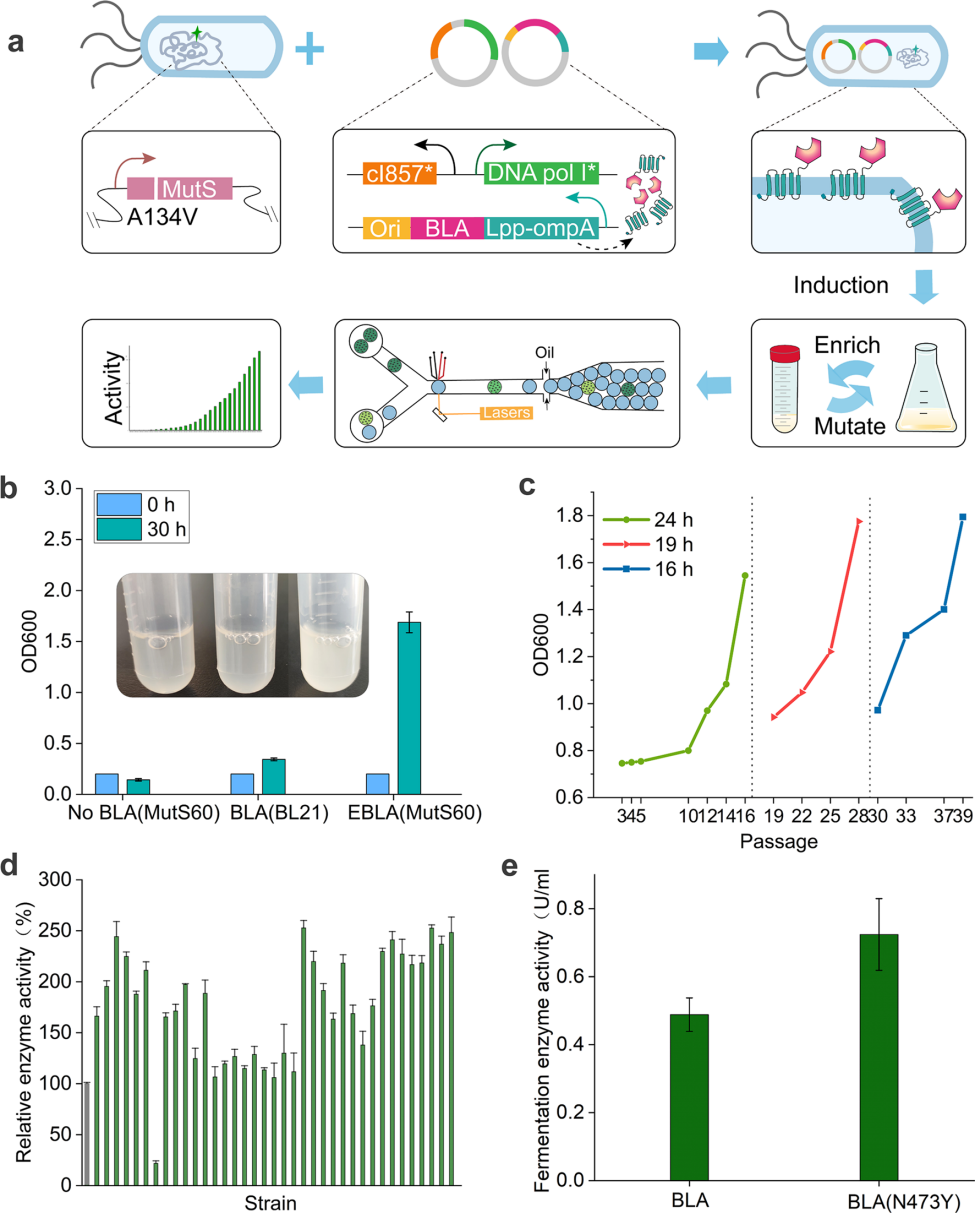
BLA in vivo evolution system and droplet microfluidic screening of BLA mutants with increased activity. **a** Scheme of the iterative mutagenic process. **b** Growth of the experimental strain and the control strains cultured for 30 h in liquid enrichment medium containing 2% starch as the sole carbon source after mutagenesis, and the initial OD_600_ was unified to 0.2. No BLA(MutS60), MutS60 strain harboring BLAdel-ori plasmid and Pol I* plasmid; BLA(BL21), BL21(DE3) strain harboring BLA-ori plasmid and no Pol I plasmid; EBLA(MutS60), MutS60 strain harboring BLA-ori plasmid and Pol I* plasmid. **c** Growth of the mutagenic cultures in the enrichment medium for 24, 19, and 16 h, respectively. **d** Relative enzyme activity of wild type (grey column) and variants (green columns) sorted by microfluidic devices. **e** Enzyme activity of the retransformed strains carrying mutant BLA (N473Y) and wild-type BLA after fermentation for 10 h in shake flasks

To express α-amylase on *E. coli* BL21 (DE3) cell surface, the carboxyl end of the outer membrane chimeric protein Lpp–OmpA was fused with the α-amylase (BLA) gene from *Bacillus licheniformis* under the control of the rhaBAD promoter induced by rhamnose [38]. The BLA activity of cells was much higher than that of the supernatant, indicating that most of the BLA protein was successfully fused with Lpp–OmpA and expressed on the surface of the extracellular membrane (Additional file 1: Fig. S3a). To construct the in vivo BLA continuous evolution system, the wild-type BLA fusion ORF was inserted upstream of ColE1 ori in the targeted plasmid (Fig. 4a). The different distances from ori did not substantially affect the expression and activity of BLA (Additional file 1: Fig. S3b). Then, this plasmid (BLA-ori) was cotransformed into cells with the mutator plasmid. BLA evolutionary library were generated through as described in the part of methods. As shown in Fig. 4b, the OD_600_ of cultures after 1 round of mutagenesis–enrichment was significantly higher than that of the control strains, reaching up to 1.5 at 30 h. Furthermore, as the number of consecutive passages increased, the growth rate in enrichment medium showed a gradual increasing trend (Fig. 4c). This indicated that the in vivo evolution system could effectively enrich for mutants with increased BLA enzyme activity.

Another challenge for in vivo evolution is identifying desired variants occurring with a lower probability in a very large library consisting mainly of individuals that are unmutated wild type. It is typically tested and analysed manually leading to low throughput and screening inefficiency. An ultrahigh-throughput screening method was established to rapidly screen strains with high BLA yields as described in previous work [39]. Single cells from the cultures through 25 round of mutagenesis–enrichment and DQ starch substrate were coembedded into microfluidic droplets. After 8 h of incubation at 25 ℃, some droplets showed stronger fluorescence, indicating that there were differences in the activity of α-amylase generated by cells in the droplets (Additional file 1: Fig. S3c and d). Subsequently, 0.1% of droplets with fluorescence signals exceeding the set threshold were sorted from approximately 3,000,000 droplets in a short time. Seventy-two percent of sorted cells showed over 20% increased activity compared with the wild-type stain (Fig. 4d). Data analysis revealed that the fermentation activity of retransformed strain carrying mutant BLA (N473Y) increased by 48.3%, due to a nucleotide change at 217 bp downstream of ColE1 ori (Fig. 4e). Further analysis showed that the combined effects of modestly increased specific enzyme activity and stability may contribute to the overall improvement in enzymatic activity (Additional file 1: Fig. S4).

### In vivo evolution of the resveratrol biosynthesis pathway in engineered *E. coli*

**Fig. 5:**
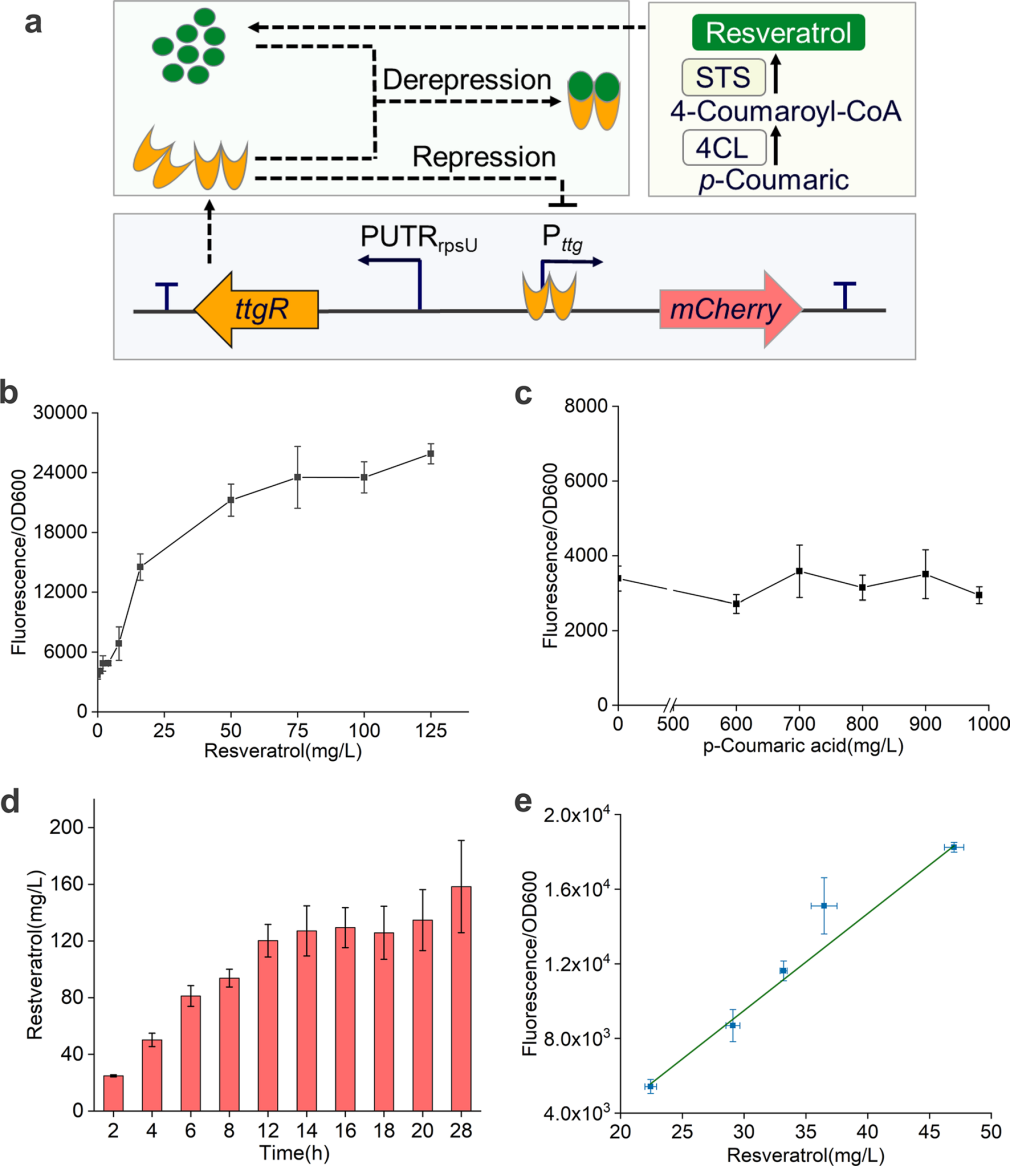
Resveratrol-responsive biosensor in *E. coli*. **a** Schematic diagram of the construction of the resveratrol biosensor and high-throughput screening method. TtgR protein specifically responds to resveratrol produced by exogenous biosynthetic pathway, thus controlling the expression of mCherry. STS, stilbene synthase; 4CL, *p*-Coumarate:CoA ligase. mCherry fluorescence of strain BL21 (DE3) harboring the pTtgR construct induced by varying concentrations of *p*-Coumaric acid (**b**) and resveratrol (**c**). **d** Resveratrol production of strain BL21 (DE3) harboring pETDuet–P_gap_–STS–4CL plasmid and pTtgR construct. **e** Characterization of the biosensor response to intracellular resveratrol biosynthesis in strain MM. Strains were cultured with exogenous addition of 900 mg/L *p-*coumaric acid at 30 °C

Resveratrol has attracted interest in the food additive, nutraceutical and pharmaceutical industries due to its various health-promoting activities. Microorganisms engineered to produce biosynthetic resveratrol with higher titres and lower costs have long been highly desired [40]. *p-*Coumarate:CoA ligase (4CL) and stilbene synthase (STS) have been reported as critical enzymes in the biosynthesis of resveratrol. To realize the in vivo evolution of these key enzymes, a biosensor-based FACS method was established (Fig. 5a).

TtgR–P_ttgABC_ system, which is responsible for multidrug recognition and especially efficient in responding to different flavonoids, is a native transcription regulatory system from *Pseudomonas putida* DOT–T1E [41, 42]. In the absence of an inducer, the transcriptional repressor TtgR binds to the P_ttgABC_ promoter and inhibits the transcription of *ttgABC* genes. When inducers are available, TtgR is released from the binding site, and downstream genes transcription is activated. To introduce the TtgR-based resveratrol biosensor to investigate the resveratrol titer, the *ttgr* gene was constructed downstream of the constitutive PUTR rpsU, and mCherry expression was placed under the control of the P_ttg_ promoter to generate the plasmid pRSFDuet–TtgR–P_ttg_–mCherry (pTtgR) (Fig. 5a). To verify the response intensity and range, different concentrations of *p-*coumaric acid and resveratrol were added for fluorescence detection. The induction was much lower in response to *p-*coumaric acid than to resveratrol, which demonstrated the excellent specificity of this sensor for resveratrol (Fig. 5b and c). Then, 4CL1 from *Arabidopsis thaliana* and STS from *Vitis vinifera* were introduced for resveratrol biosynthesis using *p*-coumaric acid as a precursor in *E. coli* BL21 (DE3). The biosynthetic pathway pETDuet–P_gap_–STS–4CL plasmid was coexpressed with the pTtgR construct. After shake flask fermentation for 28 h in the presence of 900 mg/L* p*-coumaric acid, the resveratrol titer was approximately 160 mg/L (Fig. 5d). To verify the response range of the resveratrol biosensor, plasmids Pol I*, pETDuet–ori–P_gap_–STS–4CL and pTtgR were cotransformed into the MutS60 strain to obtain the MM strain. The fluorescence intensity was positively correlated with the titer of resveratrol, which exhibited that the biosensor could be used in resveratrol biosynthesis pathway evolution (Fig. 5e).

A mutagenesis library of strain MM was constructed through iterative mutagenesis according to the developed in vivo evolution system. Given that the increased off-target mutagenesis might bring impacts on resveratrol biosensor and effective selection, stability evaluation of biosensor was performed for strains by mCherry fluorescence after 7 and 16 consecutive mutagenesis passages (Additional file 1: Fig. S5). The detection range and response trend of resveratrol biosensor did not be led to substantial change after consecutive mutagenesis passages. Accordingly, this biosensor can be used for high-throughput screening and evolution through continuous passages.

**Fig. 6:**
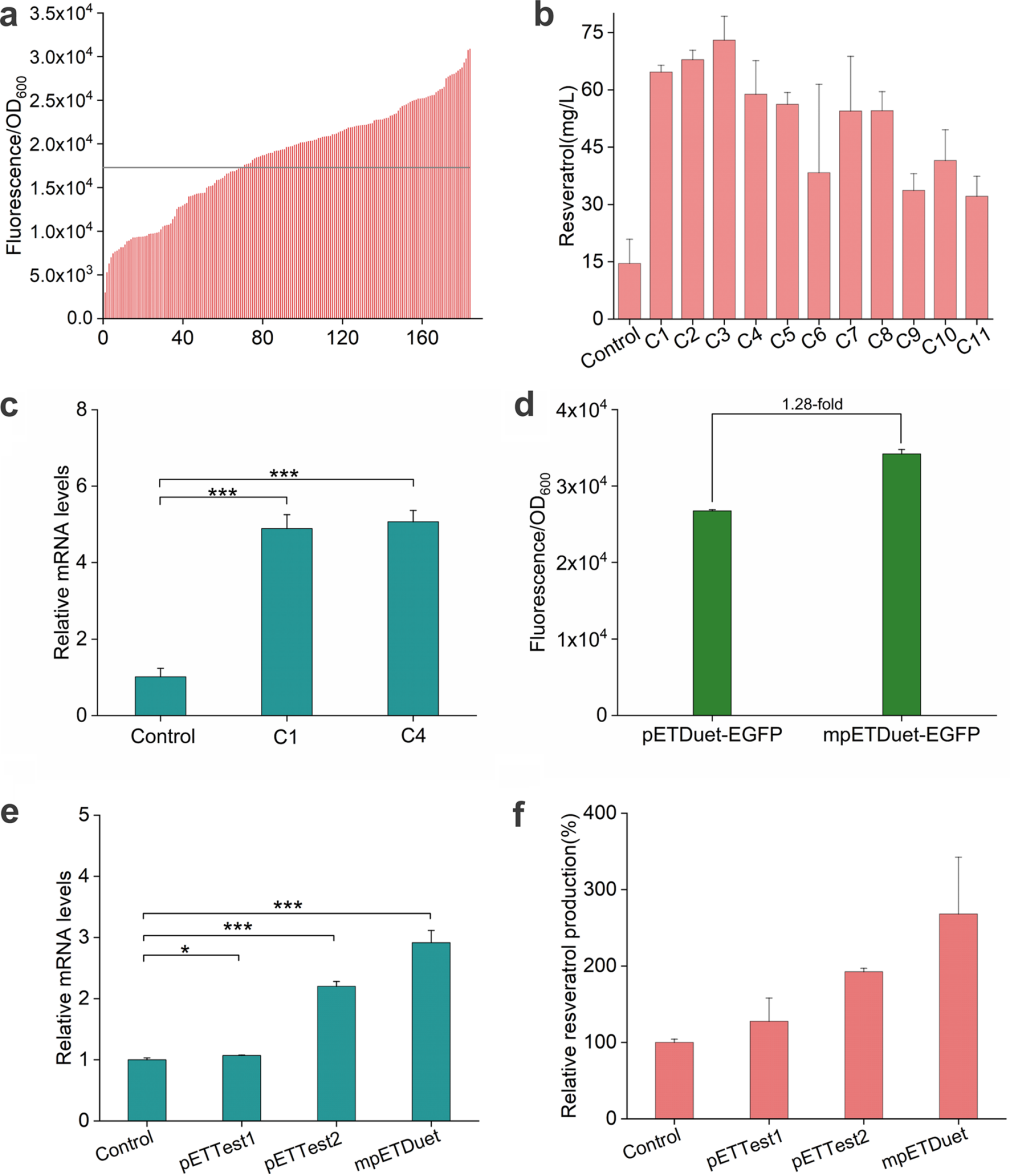
Ultrahigh-throughput screening of improved resveratrol producing strains. **a** Fluorescence intensity of strains sorted by flow cytometry. Strains were cultured in 24 deep well plates containing 900 mg/L of *p-*coumaric acid. Black line represents the fluorescence intensity of the starting strain. **b** Resveratrol productions of retransformed strains cultured in shake flasks at 30 °C. **c** RT-qPCR analysis of the control strain or cells carrying the mpETDuet plasmid expressing STS. **d** Fluorescence intensity of strain BL21 (DE3) harboring pETDuet or mpETDuet plasmid expressing EGFP. RT-qPCR analysis (**e**) and resveratrol production assay (**f**) of strains harboring the test plasmids. *P < 0.05, **P < 0.01, ***P < 0.001; two-sided student’s *t* test

After 16 consecutive mutagenesis passages, the cells with high fluorescence intensity were sorted from the saturated cultures by flow cytometry. A total of 5,000 cells were collected at a 0.592‰ sorting threshold. Over 60% of the cells sorted from the libraries exhibited improved fluorescence intensity compared with the starting strain (Fig. 6a). The plasmids were extracted from the 11 strains with the highest fluorescence intensity and transformed into fresh wild-type BL21 (DE3) cells. The resveratrol titer of the selected strains was more than twice that of the control group (Fig. 6b). The full lengths of all 11 mutants were sequenced. Interestingly, two mutations, the ColE1 ori C148T mutation and the Δ5526-6717 deletion, were identified in all plasmids; this double mutant was named mpETDuet. To verify the effect of the above mutations on the expression of STS and 4CL, the relative mRNA level of STS in retransformed stains harboring mpETDuet was approximately 4 times higher than that in the wild type (Fig. 6c). Then, the *sts* and *4 cl* genes were replaced with the *egfp* gene in the mpETDuet plasmid. As expected, the fluorescence intensity of mpETDuet was increased, by 28% (Fig. 6d).

To determine whether the improvements in resveratrol titer were associated with the mutations in the plasmid, two additional plasmids were constructed: pETTest1, containing only the ColE1 ori C148T mutation, and pETTest2, carrying only the Δ5526-6717 deletion (Additional file 1: Fig. S6). The mRNA level and resveratrol production from these plasmids were measured. The *sts* mRNA levels of pETTest1 and pETTest2 were increased by approximately 7% and 120%, respectively, compared with those of the WT. Similar to the data shown in Fig. 6c, the double mutant (mpETDuet) was 2.9-fold mRNA level of the control (Fig. 6e). Meanwhile, resveratrol production showed an increasing trend coincident with that at the mRNA level. These results suggested that the Δ5526-6717 deletion contributes greatly to the enhancement of mRNA transcription and resveratrol production (Fig. 6f). It has been reported that the *rop* gene, encoding the Rop protein, could significantly affect the plasmid copy number [43]; this gene location was coincidentally concluded in the Δ5526-6717 deletion region in our study.

## Discussion

Given the disruptions of genomic stability that occur during evolution, it is very important to employ a dynamic regulation strategy to efficiently shift between high and low mutation rates. We placed error-prone DNA Pol I* under the control of a thermoregulated expression system triggered by the evolved cI857 in *E. coli* BL21 (DE3), which permitted DNA Pol I* overexpression upon a temperature upshift. At the same time, engineered genomic MutS60 exhibited an almost normal spontaneous mutation rate at 37 °C but an increased level of mutation with increasing temperature. After evolution, a temperature downshift shifted the mutagenic process to a high-fidelity mode to maintain the stability of the desired phenotype and the host genome. Therefore, the advantage of the in vivo mutagenesis system developed in this study is that it does not require the addition and removal of inducers, making multiple rounds of iterative mutagenesis easy, rapid and controllable. However, Pol I* may be involved in parts of genome replication whose fidelity is concurrently affected by the defective mismatch repair machinery, which could give rise to off-target mutations in the *E. coli* genome during iterative mutagenesis. The resulting undesirable mutations could occur in genes essential for cell survival and affect screening for beneficial mutations [44]. Additionally, temperature upshift may generate genomic mutations to increase the fitness of library members [45].

Because the activity of Pol I* is non-predominant in the presence of genomic Pol I, the in vivo mutagenesis rate in this study is approximately 6.77 × 10^–8^ per base per generation, which is still 3–4 orders of magnitude lower than that of currently available advanced in vivo mutagenesis tools [6]. This limited mutational density had a negative impact on the exploration of sequence space and evolution speed. Another challenge for directed evolution is identifying desired variants occurring with a lower probability in a very large library consisting mainly of individuals that are unmutated wild type. It is usually difficult to establish a link between cell growth or fluorescence and the required protein function, leading to low throughput and screening inefficiency. In previous studies, most in vivo directed evolution systems were screened with the use of resistance genes and engineered auxotrophic genes, which seriously limited their broader application. In this study, we demonstrated the feasibility of using FACS and microfluidics technology-assisted ultrahigh throughput in vivo directed evolution to obtain strains with improved acid α-amylase activity and increased resveratrol production. Coincidentally, Rosenthal et al. recently reported a similar work about the controlled continuous evolution of enzymatic activity with ultrahigh throughput screening using drop-based microfluidics; these authors obtained an alditol oxidase evolved to change its substrate specificity towards glycerol [46].

Interestingly, two mutations in the high resveratrol-producing strain appeared outside key enzyme genes, including a ColE1 ori interior mutation and even a deletion mutation considerably downstream of ori. In this study, the targeted mutation rate of the wild-type strain by introduced Pol I* was still higher than that of the strain containing Pol I at a distance exceeding 1,000 bp from ori, despite the polymerase switching close to ori (~ 200 bp) [29]. These results may be attributed to the gradual replacement of Pol I by Pol III, which brings about an unbalanced mutational density [30]. At the end of lagging-strand replication, a gap of several hundred nucleotides generated by Pol III replication at 17 bp upstream of ori is likely to be associated with error-prone Pol I replication [29, 47], which may contribute to the C148T mutation in ori. However, another ~ 1,200 bp deletion mutation appeared at 3,296 bp downstream of ori. On the one hand, the deletion included the primosome assembly signal (pas) site, probably resulting in more durable Pol I replication and mutation in sequences located further from ori [15]. On the other hand, it has been reported that both Pol I and mismatch repair-defective cells may lead to large indels, which probably results in this large fragment deletion [48, 49]. In addition, the deletion mutation contains the gene encoding the Rop protein. Our data confirmed that the mutations upregulated the transcription of key enzymes, thus facilitating resveratrol production.

Furthermore, the above results indicate that this in vivo mutagenesis system has some thought-provoking limitations. With respect to the specific region within genes of interest, in vivo mutagenesis, as demonstrated in this study, presented similar issues as the current developed in vivo mutagenesis methods, such as Pol I-mediated mutagenesis [15] and OrthoRep [6]. The resulting mutants could bring desired phenotype changes unrelated to significant improvements in target protein properties, such as mutations occurring in the promoter, ribosome binding site and other regions associated with gene transcription and expression regulation, and thereby mask other variants carrying mutations within the protein-coding sequence, which is not conducive to exploring protein functional mechanisms. However, a limited mutagenesis window length restricts the molecular evolution of large protein-coding genes and long biosynthetic pathways. Thus, a long and tunable mutagenesis region is an indispensable feature of a powerful in vivo mutagenesis tool. Generally, our work could provide some reference and inspiration for the further development of in vivo continuous mutagenesis tools with high performance to accelerate protein evolution.

## Conclusions

In this study, a in vivo continuous evolution system was demonstrated temperature-controlled induction that increased the targeted mutation rate by over 600-fold, by introducing temperature-controlled error-prone DNA Pol I and a temperature-sensitive genomic MutS defect mutation. To further improve evolutionary efficiency, ultrahigh-throughput screening methods using fluorescence were used to select mutants with the desired α-amylase and resveratrol biosynthesis phenotypes. Finally, the variants with 48.3% increase in α-amylase activity and 1.7-fold higher resveratrol production were achieved. These findings demonstrated that thermal-responsive ultrahigh-throughput in vivo directed evolution can be a promising strategy for accelerating protein engineering and microbial cell factory construction.

## Methods

### Plasmids and strain cultivation

Additional file 1: Table S2 lists the strains and plasmids used in this study. All target DNA sequences were PCR-amplified by primers for plasmid construction through Gibson Assembly (Additional file 1: Tables S3 and S4). *E. coli* JM109 and BL21 (DE3) were used for gene cloning and protein expression, respectively. Transformants were grown on LB medium consisting of 5 g/L yeast extract, 10 g/L tryptone, 10 g/L NaCl and 15 g/L agar. Mutagenesis cultivation was carried out in 2*YT medium containing 10 g/L yeast extract, 16 g/L tryptone and 5 g/L NaCl. The TB medium used for fermentation contained 24 g/L yeast extract, 12 g/L tryptone, 12.54 g/L K_2_HPO_4_, 2.31 g/L KH_2_PO_4_ and 5 g/L glycerol. The medium used for enrichment of strains with enhanced α-amylase activity was composed of 12.54 g/L K_2_HPO_4_, 1.5 g/L (NH_4_)_2_SO_4_, 2.31 g/L KH_2_PO_4_ and 10 g/L soluble starch [39]. The YM9 medium for resveratrol biosynthesis consisted of 1*M9 salts, 10 g/L yeast extract, 42 g/L morpholinepropanesulfonic acid and 3% glycerol. The antibiotics added to medium and their final concentrations were as follows: 30 µg/mL chloromycetin, 100 µg/mL carbenicillin, 50 µg/mL kanamycin, 50 µg/mL spectinomycin, 100 µg/mL ampicillin and 100 µg/mL rifampicin.

### cI857 library construction and screening

The cI857 random mutant library was constructed using the GeneMorph II Random Mutagenesis Kit (Agilent Technologies, CA, USA), and assembled into plasmid pRSFDuet–P_asnS_–cI857 to replace the wild-type cI587, resulting in the cI857 plasmid library. Thereafter, cI857 library was transformed into *E. coli* BL21 (DE3) harboring plasmid pACYCDuet–λPR–EGFP. After recovery for 1 h at 30 ℃, the transformants approximately 1 mL in total were completely transferred to a sterile test tube supplemented with 1 mL fresh liquid LB, and grown for 8 h in a 37 °C shaker to induce EGFP expression, instead of spreading plates. Subsequently, cI857 library cells are centrifuged to remove the supernatant and washed three times with PBS buffer. The cells were then resuspended and diluted to OD_600_ of 0.1 in PBS buffer, and sorted using a flow cytometer FACSArica III (BD Biosciences, CA, USA).

The first round of FACS was performed for positive screening, and the top 0.3% of cells with highest fluorescence intensity were collected in 2 mL LB. The collected cells were then cultured for 12 h in a 30 °C shaker, and prepared for second round negative screening. In the second round of FACS, the bottom 0.4% of cells with lowest fluorescence intensity were collected in 2 mL LB. After two rounds of FACS, the collected cells were coated on LB plates and cultured at 37 ℃. 768 single colonies shown darkest green were picked into 96-shallow-well plates at 30 ℃. After 8 h of growth, the cultures were transferred at 5% inoculum into 800 μL TB divided into 96-deep-well plates, and cultured respectively for 9 h at 30 ℃ and 37 ℃ after being grown for 3 h at 30 °C. Subsequently, the fluorescence assay of cells washed and resuspended in the PBS was performed on a microplate reader (BioTek, California, USA) (excitation at 488 nm and emission at 520 nm). To further characterize the induction performance, single colonies were grown overnight in 20 mL LB at 30 ℃, and then transferred at 1% inoculum to shake flasks containing 25 mL TB. Until OD_600_ was up to 0.6–0.8, the cultures were induced respectively at 30 ℃ and 37 ℃. The EGFP fluorescence and OD_600_ was detected. The fluorescence intensity subtracting background fluorescence was calculated for mutant strains, and starting strain as a control.

### Determination of reversion frequency and mutation rate

The mutator plasmid (no Pol I/Pol I/Pol I* plasmid) and target plasmid (pTA or pTS 250/500/1000/2000/2500/3000 plasmid) were cotransformed into *E. coli* BL21 (DE3), and cultured on LB plates overnight at 30 ℃. Seed and mutagenesis cultivation were carried out using the optimized method previously [15]. For mutagenesis cultivation, cultures were transferred at 1:10^5^ dilutions into shake flasks containing 50 mL 2*YT medium, then grown to saturation at 37 ℃. Subsequently, the dilutions of cultures then were coated on LB plates containing chloramphenicol and kanamycin. For the count of gain-of-resistant colonies, cells were plated on LB plates with 100 µg/mL carbenicillin or 50 µg/mL spectinomycin. The reversion frequency was determined by the ratio of the number of carbenicillin or spectinomycin-resistant colonies to the total of viable colonies. The mutation rate was then determined according to previously described method [15].

### Genomic modification of mismatch repair protein MutS

MutS A134V mutation was introduced into *E. coli* BL21 (DE3) genome using CRISPR–Cas9 system [50]. The homologous sequence containing A134V was constructed and 20 nucleotides near the A134V mutation site were replaced with synonymous codons (except rare codons). The correct transformant was obtained by colony PCR and Sanger sequencing. Plasmid sgRNA and pCas9 were sequentially eliminated by culturing in LB containing 0.5 mmol/L IPTG at 30 ℃ and serial passaging in antibiotic-free LB medium at 37 ℃, resulting in strain MutS60.

### Protein expression and α-amylase enzyme activity assay

Plasmid pET28a–P_rhaBAD_–lpp–ompA–BLA was transformed into *E. coli* BL21 (DE3). Single colony was cultured overnight at 30 ℃. Cultures were transferred at 1% inoculum to shake flasks containing 25 mL LB. When OD_600_ reached 0.6, a final concentration of 1 mmol/L rhamnose was added into medium to induce protein expression at 25 ℃. After 10 h of cultivation, the α-amylase activity at 70 ℃ in the cells and supernatant was determined according to the 3,5-dinitrosalicylic acid (DNS) method [39].

For α-amylase purification, plasmid pET28a–P_rhaBAD_–BLA–6*His tag-ori was transformed into *E. coli* BL21 (DE3). Cells from the fermentation cultures were centrifuged, resuspended and lysed at 800 bar using a high pressure homogenizer (Union biotech, Shanghai, China). The lysis supernatant was filtered with 0.22 μm mixed cellulose membrane and purified using HisTrap™ HP column (GE Healthcare) by affinity chromatography. Thereafter, the solution eluted by the buffer (150 mM imidazole, 50 mM Tris–HCl and 100 mM NaCl) was collected and removed imidazole by ultrafiltration. Finally, the eluent was analysed by sodium dodecyl sulfate–polyacrylamide gel electrophoresis (SDS–PAGE) and protein concentration was determined using the previously described method [51].

### In vivo continuous evolution and microfluidic screening of α-amylase

Gene expression cassette (P_rhaBAD_–lpp–ompA–BLA) for α-amylase was cloned into vector pET28a to assemble plasmid pET28a–P_rhaBAD_–lpp–ompA–BLA-ori (BLA-ori). The mutator plasmid Pol I* and target plasmid BLA-ori were cotransformed into strain MutS60. For mutagenesis, seed cultures were transferred at 1:10^5^ dilutions into 50 mL 2*YT medium and grown to saturation at 43 ℃. For enrichment, the saturated cultures were collected and washed 3 times in the PBS. The cells were diluted to OD_600_ of 0.2 in the enrichment medium, and cultured until OD_600_ exceeded 0.8 at 30 ℃. Cultures were then diluted into 50 mL 2*YT and passaged for the next round of mutagenesis and enrichment.

The cultures through mutagenesis-enrichment process were transferred at 4% inoculum into 25 mL LB. After induced protein expression for 1.5 h at 30 ℃, the library cells were washed twice in the PBS and diluted to OD_600_ of 0.01 in LB containing 0.05 mmol/L rhamnose, 50 µg/mL kanamycin and 30 µg/mL chloromycetin as a water phase. DQ starch substrate (Invitrogen, Eugene, OR) was diluted to 100 μg/mL as another water phase. By employing the microfluidic droplet generator, the two separate water phases with the flow rate of 1 μL/min were co-embedded using 2% surfactant Pico Surf (Sphere fluidics, UK) in HFE 7500 (3 M, UK) as the oil phase with a flow rate of 5 μL/min. Single cell droplets were then collected and incubated in a 1 mL syringe at 25 or 30 ℃. Subsequently, the droplets exceeding the fluorescence threshold were sorted out by the sorting device. The sorted droplets were broken by adding demulsifiers and vortex oscillation. The cells were then extracted and spread on LB plates after mixing the emulsion and 300 µL fresh LB.

### Characterization of resveratrol biosensor

Plasmid pCDFDuet–TtgR–P_ttg_–mCherry (pTtgR) was transformed into *E. coli* BL21 (DE3). Different final concentrations of *p*-coumaric acid and resveratrol were added into LB medium for 24-deep-well-plate fermentation at 30 ℃. The mCherry fluorescence was detected (excitation at 580 nm and emission at 610 nm) after 4 h. For the biosynthesis of resveratrol, plasmid pETDuet–Pgap–STS–4CL was transformed into *E. coli* BL21 (DE3) [52]. Single colonies were grown overnight at 30 ℃, and transferred at 1% inoculum into 25 mL YM9. When OD_600_ reached 0.8–1, 900 mg/L *p*-coumaric acid as precursor was added into medium to produce resveratrol. Subsequently, resveratrol was detected at 305 nm on a Prominence LC-20A instrument (Shimadzu, Kyoto, Japan) equipped with a Hypersil™ ODS-2 C18 column (250 × 4.6 mm, 5 µm) (Thermo Fisher Scientific, Waltham, MA, USA) according to previously reported method [53].

### Iterative mutagenesis and high-throughput screening of resveratrol producing strains

Strain MutS60 harboring plasmid Pol I*, pETDuet–ori–Pgap–STS–4CL and pTtgR was grown to OD_600_ of 0.5 at 30 ℃. For mutagenesis, seed cultures were transferred at 1:10^5^ dilutions into 50 mL 2*YT medium and grown to saturation at 43 ℃. The saturated cultures were then passaged for next mutagenesis. Before preparing for sorting, cultures from 16 passages were transferred at 2% inoculum into 25 mL YM9 at 30 ℃. When OD_600_ was 0.8–1, 900 mg/L* p*-coumaric acid was added into medium for 10 h of cultivation. Subsequently, sample was harvested, washed, and resuspend in the PBS for FACS. The collected cells were incubated on LB plates containing 900 mg/L *p*-coumaric acid. About 170 single colonies with darkest red were selected for 24-deep-well-plate fermentation at 30 ℃. Plasmids were extracted from the top 11 strains with highest fluorescence intensity, and retransformed into wild-type *E. coli* BL21 (DE3) to verify the increase in resveratrol production.

### RT–qPCR analysis

The relative mRNA level of STS gene in retransformed strains were measured by RT-qPCR, taken *rrs*A gene encoding ribosomal RNA 16S as reference gene. Specifically, after 10 h of fermentation, 1 mL sample was harvested for preparation of total RNA and RT–qPCR analysis as previously described method [54].

### Supplementary Information

**Additional file 1: Fig. S1.** Characterization of P_R_-cI857 or evolved P_R_-cI857* expression system. The fluorescence intensity of mutant M1 and wild-type strain after 3 h (**a**) and 6 h (**b**) of fermentation in the shake flask at 30 ℃/37 ℃. **c** Mutator plasmid profile carrying *egfp* gene under the control of P_R_-cI857 or evolved P_R_-cI857* expression system. **d** Fluorescence intensity of strains after cultivation for 6 h at 30 ℃/37 ℃ in TB media. **Fig. S2.** Reversion frequency of strains. **a** Reversion frequency of strains harboring the reporter plasmid pTS. **b** Growth curve of strain harboring the target plasmid and mutator plasmid for mutagenesis cultivation at 37℃ in 2*YT media. The cultures through 18 h of mutagenesis cultivation were used for evaluating cell reversion rate. **c** Reversion frequency of strains harboring the reporter plasmid pTA. **d** Growth curve of strain mutS60 harboring the target plasmid and mutator plasmid for mutagenesis cultivation at 43 ℃ in 2*YT media. **Fig. S3.** Cell surface display and microfluidic screening of BLA. **a** Enzyme activity of BLA in cells and supernatant. **b** Enzyme activity of BLA at different distances from Ori. During the ColE1 replication process, the position where the first incorporated deoxyribonucleotide was defined as + 1. BLA: + 1173; BLA near ori: + 1. **c** Schematic illustration of droplet generation. **d** Fluorescence of microfluidic droplets incubated for several hours at 25 ℃/30 ℃. **Fig. S4.** Stability of wild-type (WT) BLA and mutant BLA(N473Y). a. SDS–PAGE of the purified enzymes. b. Relative specific activity of the purified enzymes. **Fig. S5.** mCherry fluorescence intensity of strain MM with exogenous addition of resveratrol. B0: starting strain MM; B7: strain MM after consecutive mutagenesis passages for 7 times; B16: strain MM after consecutive mutagenesis passages for 16 times. **Fig. S6.** Test plasmids construction for analysis of mutations in the mpETDuet plasmid. Left: control plasmid containing the original sequence. Middle: test plasmids containing either ColE1 ori C148T mutation or Δ5526-6717 deletion. Right: mpETDuet plasmid containing both ColE1 ori C148T mutation and Δ5526-6717 deletion. **Table S1.** Mutation spectrum at TAA of wild-type strain with Pol I* plasmid (aadA as reporter). **Table S2.** Plasmids and strains used in this work. **Table S3.** Oligonucleotides used in this study. **Table S4.** DNA sequences used in this study.

## Data Availability

The datasets generated or analyzed during this study are included in this published article and its supplementary materials.
