# Supplementary material for: Ultrahigh-throughput screening-assisted in vivo directed evolution for enzyme engineering
Source: Biotechnol Biofuels Bioprod. 2024 Jan 22;17:9. doi: 10.1186/s13068-024-02457-w (PMC10804518; doi:10.1186/s13068-024-02457-w)
Supplement: Supplementary file 1 — Additional file 1: Fig. S1. Characterization of PR-cI857 or evolved PR-cI857* expression system. The fluorescence intensity of mutant M1 and wild-type strain after 3 h (a) and 6 h (b) of fermentation in the shake flask at 30 ℃/37 ℃. c Mutator plasmid profile carrying egfp gene under the control of PR-cI857 or evolved PR-cI857* expression system. d Fluorescence intensity of strains after cultivation for 6 h at 30 ℃/37 ℃ in TB media. Fig. S2. Reversion frequency of strains. a Reversion frequency of strains harboring the reporter plasmid pTS. b Growth curve of strain harboring the target plasmid and mutator plasmid for mutagenesis cultivation at 37℃ in 2*YT media. The cultures through 18 h of mutagenesis cultivation were used for evaluating cell reversion rate. c Reversion frequency of strains harboring the reporter plasmid pTA. d Growth curve of strain mutS60 harboring the target plasmid and mutator plasmid for mutagenesis cultivation at 43 ℃ in 2*YT media. Fig. S3. Cell surface display and microfluidic screening of BLA. a Enzyme activity of BLA in cells and supernatant. b Enzyme activity of BLA at different distances from Ori. During the ColE1 replication process, the position where the first incorporated deoxyribonucleotide was defined as + 1. BLA: + 1173; BLA near ori: + 1. c Schematic illustration of droplet generation. d Fluorescence of microfluidic droplets incubated for several hours at 25 ℃/30 ℃. Fig. S4. Stability of wild-type (WT) BLA and mutant BLA(N473Y). a. SDS–PAGE of the purified enzymes. b. Relative specific activity of the purified enzymes. Fig. S5. mCherry fluorescence intensity of strain MM with exogenous addition of resveratrol. B0: starting strain MM; B7: strain MM after consecutive mutagenesis passages for 7 times; B16: strain MM after consecutive mutagenesis passages for 16 times. Fig. S6. Test plasmids construction for analysis of mutations in the mpETDuet plasmid. Left: control plasmid containing the original sequence. Middle: test plasmids co [file 13068_2024_2457_MOESM1_ESM.docx]

**Additional file**

**Ultrahigh-throughput screening assisted *in vivo* directed evolution for**

**enzyme engineering**

Shuaili Chen^1,2^, Zhanhao Yang^1,2^, Ze Zhong^1,2^, Shiqin Yu^1,2^, Jingwen Zhou^1,2,3,4^, Jianghua Li^1,3,4^, Guocheng Du^1,3,4^, Guoqiang Zhang^1,2,3,4^*

1. Science Center for Future Foods, Jiangnan University, 1800 Lihu Road, Wuxi, Jiangsu 214122, China.

2. National Engineering Research Center for Cereal Fermentation and Food Biomanufacturing, Jiangnan University, 1800 Lihu Road, Wuxi, Jiangsu 214122, China.

3. Engineering Research Center of Ministry of Education on Food Synthetic Biotechnology, Jiangnan University, 1800 Lihu Road, Wuxi, Jiangsu 214122, China.

4. Jiangsu Province Engineering Research Center of Food Synthetic Biotechnology, Jiangnan University, 1800 Lihu Road, Wuxi, Jiangsu 214122, China.

*Corresponding authors: Guoqiang Zhang

Mailing address: School of Biotechnology, Jiangnan University, 1800 Lihu Road, Wuxi, Jiangsu 214122, China

Phone: +86-510-85914371, Fax: +86-510-85914371

E-mail: gqzhang@jiangnan.edu.cn (Zhang GQ)

**Additional file Figures**


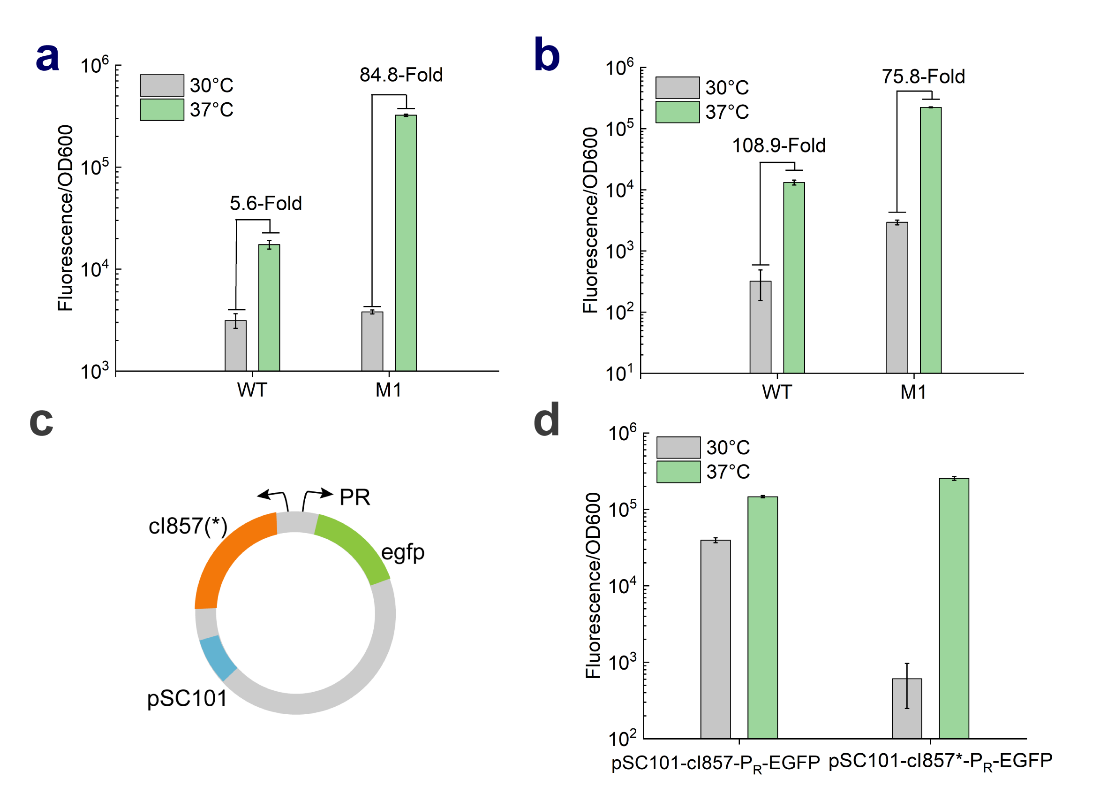


**Fig. S1 Characterization of** **P_R_-cI857 or evolved P_R_-cI857* expression system.** The fluorescence intensity of mutant M1 and wild-type strain after 3 h (a) and 6 h (b) of fermentation in the shake flask at 30℃/37℃. c. The mutator plasmid profile carrying *egfp* gene under the control of P_R_-cI857 or evolved P_R_-cI857* expression system. d. The fluorescence intensity of strains after cultivation for 6 h at 30℃/37℃ in TB media.


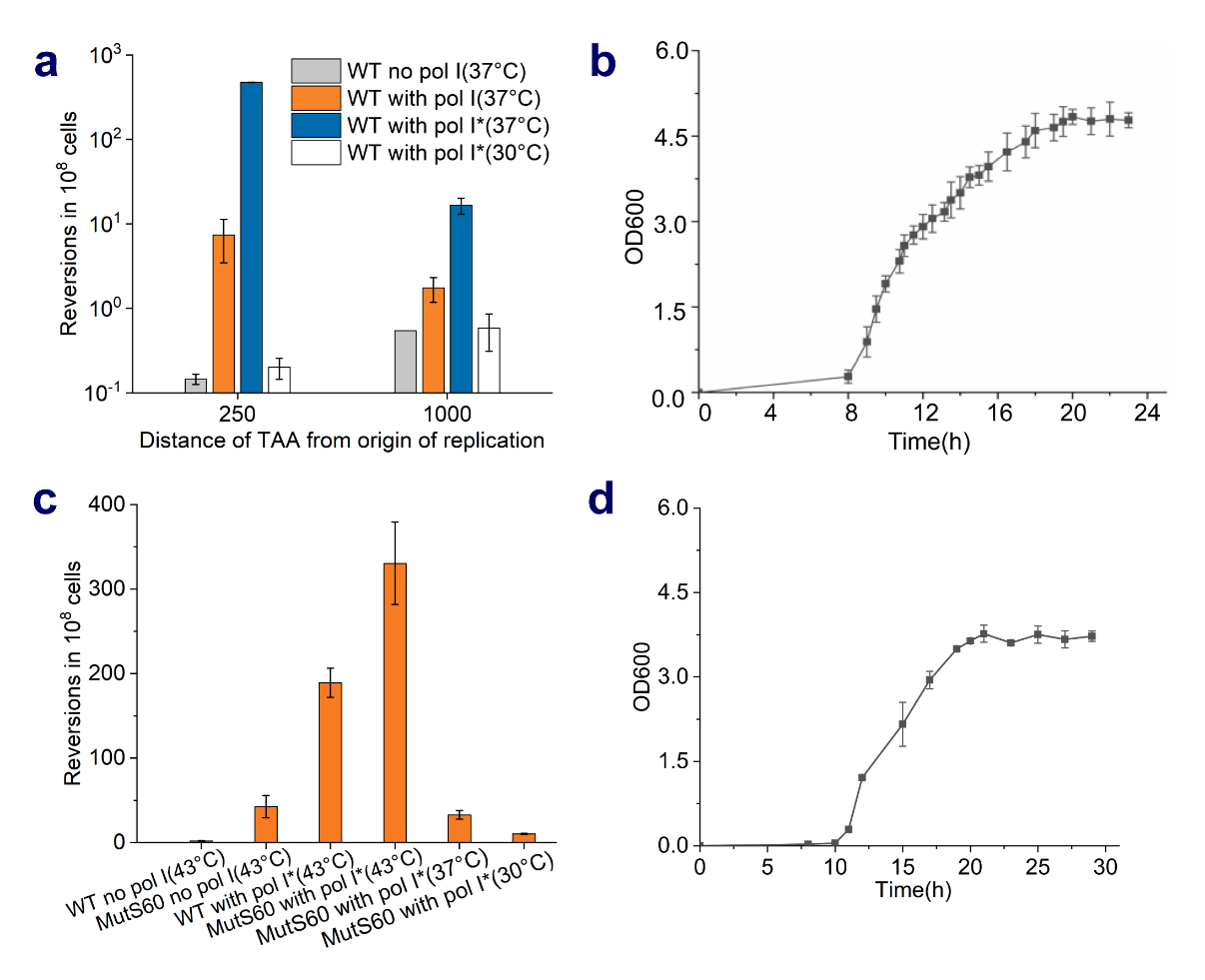


**Fig. S2** **Reversion frequency of strains.** a. The reversion frequency of strains harboring the reporter plasmid pTS. b. The growth curve of strain harboring the target plasmid and mutator plasmid for mutagenesis cultivation at 37℃ in 2*YT media. The cultures through 18 h of mutagenesis cultivation were used for evaluating cell reversion rate. c. The reversion frequency of strains harboring the reporter plasmid pTA. d. The growth curve of strain mutS60 harboring the target plasmid and mutator plasmid for mutagenesis cultivation at 43℃ in 2*YT media.


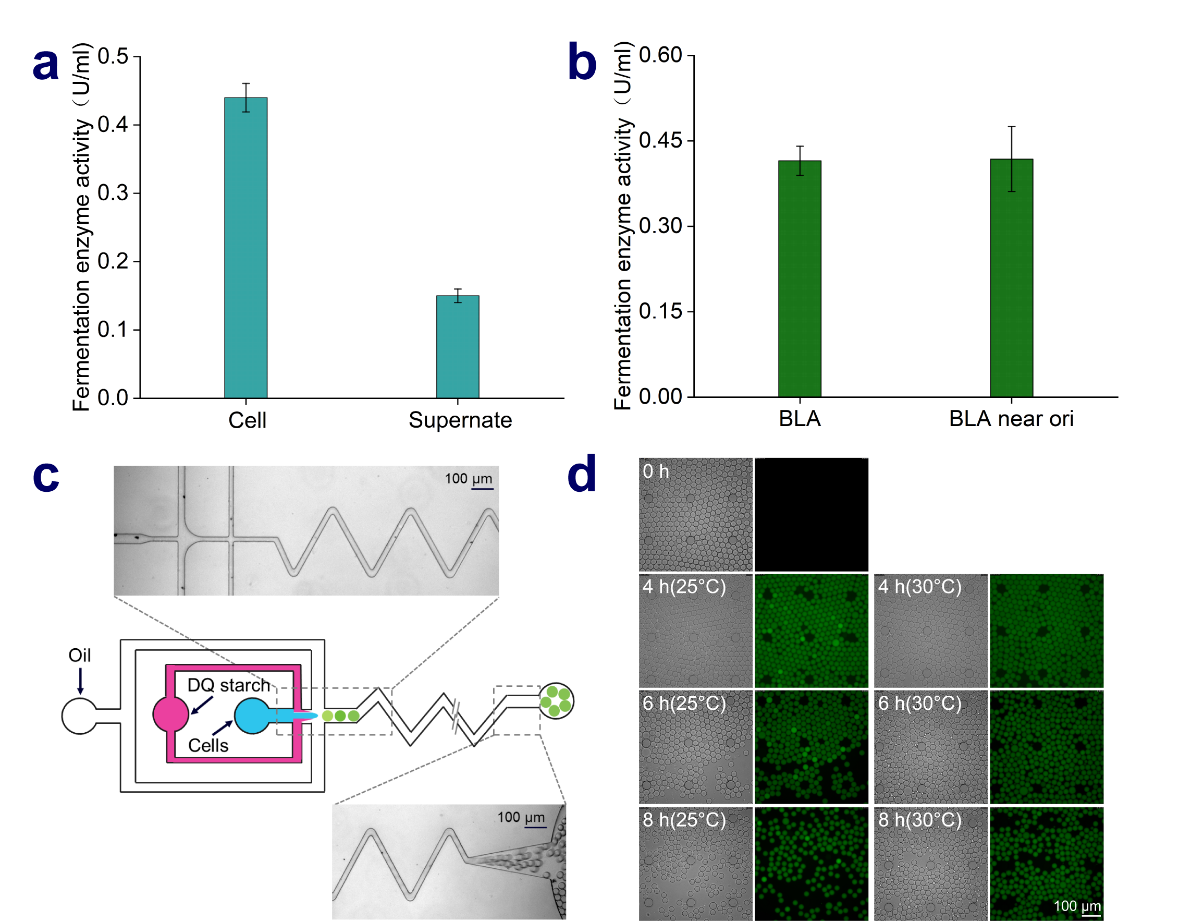


**Fig. S3 Cell surface display and microfluidic screening of BLA.** a. The enzyme activity of BLA in cells and supernatant. b. The enzyme activity of BLA at different distances from Ori. During the ColE1 replication process, the position where the first incorporated deoxyribonucleotide was defined as +1. BLA: +1173; BLA near ori: +1. c. Schematic illustration of droplet generation. d. The fluorescence of microfluidic droplets incubated for several hours at 25℃/30℃.


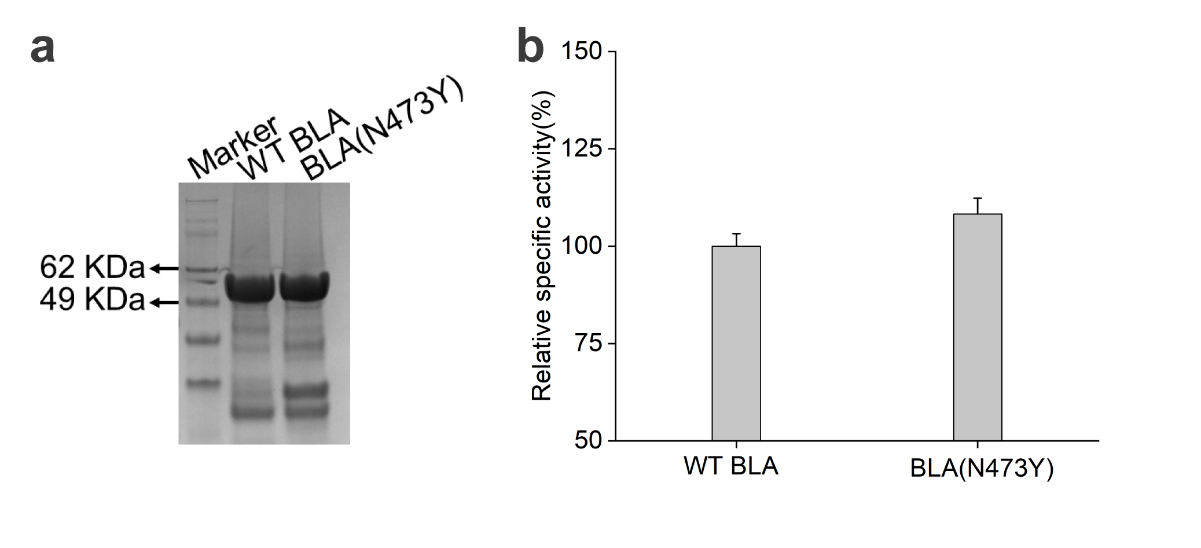


**Fig. S4 Stability of wild-type (WT) BLA and mutant BLA(N473Y).** a. SDS-PAGE of the purified enzymes. b. Relative specific activity of the purified enzymes.





**Fig. S5 mCherry fluorescence intensity of strain MM with exogenous addition of resveratrol.** B0: starting strain MM; B7: strain MM after consecutive mutagenesis passages for 7 times; B16: strain MM after consecutive mutagenesis passages for 16 times.


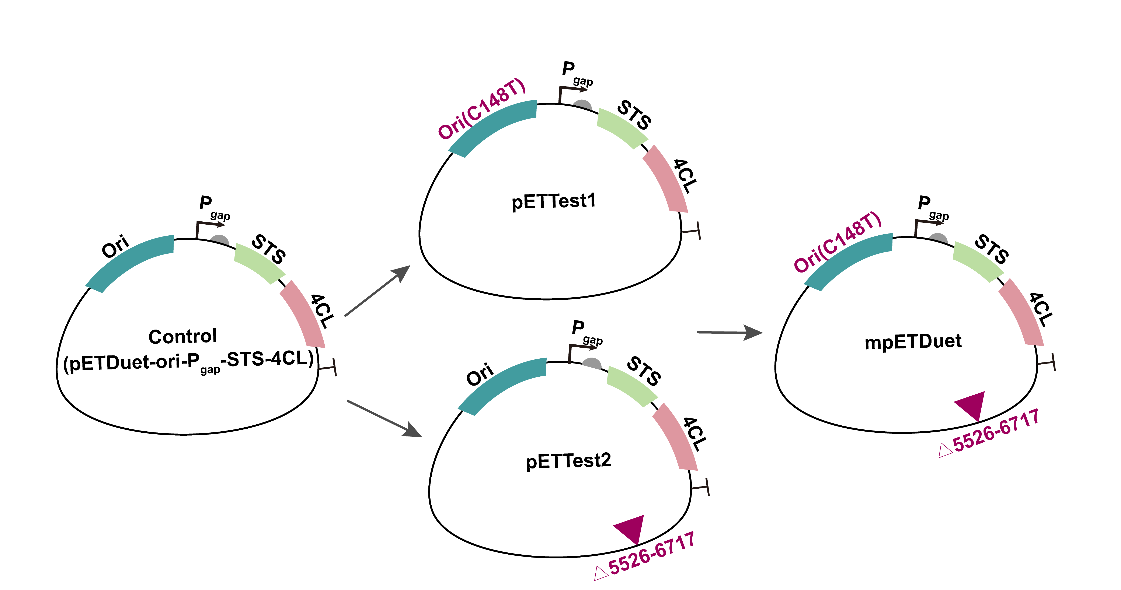


**Fig. S6** **Test plasmids construction for analysis of mutations in the mpETDuet plasmid.** Left: control plasmid containing the original sequence. Middle: test plasmids containing either ColE1 ori C148T mutation or Δ5526-6717 deletion. Right: mpETDuet plasmid containing both ColE1 ori C148T mutation and Δ5526-6717 deletion.

**Additional file Tables**

**Table S1 Mutation spectrum at TAA of wild-type strain with Pol I* plasmid (aadA as reporter)**

| Nucleotide changes | Amino acid changes | Count | Frequence |
| --- | --- | --- | --- |
| TAA→TTA | STOP→L | 50 | 100% |
| Total | —— | 50 | 100% |

**Table S2 Plasmids and strains used in this work**

| Plasmid/Strain | Description | Sources |
| --- | --- | --- |
| Plasmid |  |  |
| pSC101 | Expression vector, chloromycetin resistance | Lab stock |
| pET28a | Expression vector, kanamycin resistance | Lab stock |
| pETDuet-1 | Expression vector, ampicillin resistance | Lab stock |
| pCDFDuet-1 | Expression vector, spectinomycin resistance | Lab stock |
| pRSFDuet-1 | Expression vector, kanamycin resistance | Lab stock |
| pACYCDuet-1 | Expression vector, chloramphenicol resistance | Lab stock |
| p034-LppOmpA | Vector p034 carrying a rhamnose-inducible promoter rhaBAD, and carrying Lpp-OmpA with (G4S)_3_ linker at carboxyl end | Lab stock |
| pRSFDuet-P_asnS_-cI857 | pRSFDuet-1 carrying *cI857* gene under asnS promoter-5′-UTR complex (PUTR) | This work |
| pACYCDuet-λPR-EGFP | pACYCDuet-1 carrying *egfp* gene under λPR promoter | This work |
| pSC101-cI857-λPR-EGFP | pSC101 carrying *cI857* gene under asnS PUTR, and carrying *egfp* gene under λPR promoter | This work |
| pSC101-cI857*-λPR-EGFP | pSC101 carrying the evolved cI857 gene under asnS PUTR, and carrying *egfp* gene under λPR promoter | This work |
| pSC101-cI857*-λPR-Pol I* (Pol I*) | pSC101 carrying the evolved cI857 gene under cascade PUTR (PUTR_ssrA_-PUTR_infC‑rplT_), and carrying *pol I** (Pol I D424A I709N A759R) gene under λPR promoter | This work |
| pSC101-cI857*-λPR-Pol I (Pol I) | pSC101 carrying the evolved cI857 gene under cascade PUTR (PUTR_ssrA_-PUTR_infC‑rplT_), and carrying the *pol I* gene under λPR promoter | This work |
| pSC101-cI857*-λPR-no Pol I (no Pol I) | pSC101 carrying the evolved cI857 gene under cascade PUTR (PUTR_ssrA_-PUTR_infC‑rplT_), and carrying no gene under λPR promoter | This work |
| pTA250/500/1000/2000/2500/3000 | pET28a carrying the β-lactamase (E26Stop) gene with a premature TAA codon at 250/500/1000/2000/2500/3000bp downstream of ColE1 ori under constitutive AmpR promoter | This work |
| pTS250/1000 | pET28a carrying the *aadA* (L33Stop) spectinomycin resistance gene with a premature TAA codon at 250/1000bp downstream of ColE1 ori under constitutive AmpR promoter | This work |
| pET28a-P_rhaBAD_-lpp-ompA-BLA (BLA) | pET28a carrying the *bla* gene with N-terminal fusion expression of lpp-ompA chimeric through (G4S)_3_ linker under inducible promoter rhaBAD for BLA surface display | This work |
| pET28a-P_rhaBAD_-lpp-ompA-BLA-ori (BLA-ori) | pET28a carrying the BLA expression cassette located downstream of ColE1 ori (+1, the first deoxyribonucleotide downstream of ColE1 ori was defined as +1) | This work |
| BLAdel-ori | Derived from plasmid BLA-ori, deleted the *bla* gene | This work |
| pET28a-P_rhaBAD_-BLA-6*His tag-ori | pET28a carrying the *bla* gene with N-terminal His-tag for protein purification | This work |
| pETDuet-P_gap_-STS-4CL | pETDuet-1 carrying the *sts* gene from *Vitis vinifera* (Genbank accession No. EF192465.1), *4cl* gene from *Arabidopsis thaliana* (Genbank accession No. U18675.1) and intergenic sequence (TGAAGGCAA) under constitutive GAP promoter | This work |
| pCDFDuet-TtgR-P_ttg_-mCherry (pTtgR) | pCDFDuet-1 carrying the *ttgr* gene under constitutive PUTR rpsU, and carrying *mCherry* gene under promoter P_ttg_ | This work |
| pETDuet-ori-P_gap_-STS-4CL | pETDuet-1 carrying STS and 4CL expression cassette located downstream of ColE1 ori (+1) | This work |
| pETDuet-EGFP | Derived from pETDuet-ori-P_gap_-STS-4CL, where *egfp* gene replaced the *sts* and *4cl* gene | This work |
| mpETDuet-EGFP | Derived from pETDuet-ori-P_gap_-STS-4CL extracted from mutant strain (mpETDuet), where *egfp* gene replaced the *sts* and *4cl* gene | This work |
| pETTest1 | Derived from pETDuet-ori-P_gap_-STS-4CL, containing ColE1 ori C148T mutation | This work |
| pETTest2 | Derived from pETDuet-ori-P_gap_-STS-4CL, containing △5526-6717 deletion | This work |
| Strain |  |  |
| *E. coli* JM109 | Wild type for gene cloning | Lab stock |
| *E. coli* BL21(DE3) | Wild type for protein expression | Lab stock |
| MutS60 | *E. coli* BL21(DE3) carrying MutS A134V mutation | This work |

**Table S3 Oligonucleotides used in this study**

| Primer | Sequence |
| --- | --- |
| cI-F1 | gagaatattaaggagatataccATGAGCACAAAAAAG |
| cI-R1 | GGCAGCAGCCTAGGTTAATCAGCCAAACGTCTCTTCAGGC |
| P_asnS_-F | CACGGCCGCATAATCGAAATtctttcgctgcatttgcgaat |
| P_asnS_-R | ggtatatctccttaatattctctctgttaatagtcggaa |
| pRSFDuet-F1 | TTAACCTAGGCTGCTGCCACCG |
| pRSFDuet-R1 | TTCGATTATGCGGCCGTGTACAAT |
| λPR-F | gtgcgtgTTGACTATTTTACCTCTGGCGGTGATAATGGTTGCACCATCTTAGTATATTAGTTAAGTATAAGAAGGAGATATACATatg |
| λPR-R | GTAAAATAGTCAAcacgcacggtgttagatatttatcccttgcggtgatagatttaacgtatgATTTCGATTATGCGGCCGTGTAC |
| EGFP-F | atgggtaagggagaagaacttttcac |
| EGFP-R | TGGCAGCAGCCTAGGTTAAttatttgtatagttcatccatgccatgtgt |
| pACYC-EGFP-F | TTAACCTAGGCTGCTGCCACC |
| pACYC-EGFP-R | ttcttctcccttacccatATGTATATCTCCTTCTTATACTTAACTAATATACTAAGATG |
| cI-F2 | gagaatattaaggagatataccATGAGCACAAAAAAG |
| cI-R2 | GGCAGCAGCCTAGGTTAATCAGCCAAAC |
| pRSFDuet-F2 | ATTAACCTAGGCTGCTGCCAC |
| pRSFDuet-R2 | ggtatatctccttaatattctctctgttaatagtcggaa |
| PUTR_ssrA_-F | ctttaacacgaatgcccgcaatgtgtaaaggtaagtataccagatttatgagcg |
| PUTR_ssrA_-R | ACGATTACTTTCTGTTCGAattggctatcacatccgacacaaatg |
| PUTR_infC‑rplT_-F | GTTTCTTTTTTGTGCTCATaccttattcctccaattgtttaagactgcg |
| PUTR_infC‑rplT_-R | gcgggcattcgtgttaaagcag |
| cI-F3 | GCGACTCCTGCATTAGGAAATCAAAAAACCCCTCAAGACCCGTTTAG |
| cI-R3 | ATGAGCACAAAAAAGAAACCATTAACACAAGAG |
| λPR-EGFP-F | TCGAACAGAAAGTAATCGTATTGTACACGGC |
| λPR-EGFP-R | ttatttgtatagttcatccatgccatgtgt |
| pSC-EGFP-F | catggcatggatgaactatacaaataaTTAACCTAGGCTGCTGCCACC |
| pSC-EGFP-R | ATTTCCTAATGCAGGAGTCGCATAAGG |
| pol-F | TAAGAAGGAGATATACATatggttcagatcccccaaaatcca |
| pol-R | ttagtgcgcctgatcccagttttc |
| pSC-pol-F | ctgggatcaggcgcactaaTTAACCTAGGCTGCTGCCACC |
| pSC-pol-R | ggatctgaaccatATGTATATCTCCTTCTTATACTTAACTAATATACTAAGATGGT |
| pSC101-cI857*-λPR-empty-F | GTATATTAGTTAAGTATAAGAAGGAGATATACATATGTTAACCTAGGCTGCTGCCACC |
| pSC101-cI857*-λPR-empty-R | catATGTATATCTCCTTCTTATACTTAACTAATATACTAAGATGGT |
| lactamase-pTA-F | CTCAGCTTCCTTTCGGGCTTTGTTACCAATGCTTAATCAGTGAGGCACC |
| lactamase-pTA-R | ATCTCGATCCCGCGAAATCGCGGAACCCCTATTTGTTTATTTTTC |
| pET28a-pTA-F | ATTTCGCGGGATCGAGATCTCG |
| pET28a-pTA-R | CAAAGCCCGAAAGGAAGCTGAG |
| E26*-F | ACCAGCGTTTATGGGTGAGCAAAAACAGGAAGGCAA |
| E26*-R | GCTCACCCATAAACGCTGGTGAAAGTAAAAGATGCT |
| lactamase-ORF-F | CAAAAAACCCCTCAAGACCCGTT |
| lactamase-ORF-R | CGCGGAACCCCTATTTGTTTATTTTTC |
| pET28a-pTA250-F | ACAAATAGGGGTTCCGCGAGGAAAGAACATGTGAGCAAAAGGC |
| pET28a-pTA250-R | GGTCTTGAGGGGTTTTTTGGCGTTATCCCCTGATTCTGTGG |
| pET28a-pTA500-F | ACAAATAGGGGTTCCGCGGACCCAGTCACGTAGCGATAGCGG |
| pET28a-pTA500-R | GTCTTGAGGGGTTTTTTGATGGCTGCGCCCCGACAC |
| pET28a-pTA1000-F | AAACAAATAGGGGTTCCGCGGTATTAACGAAGCGCTGGCATTG |
| pET28a-pTA1000-R | GGTCTTGAGGGGTTTTTTGAGATGTAGGTGTTCCACAGGGTAG |
| pET28a-pTA2000-F | ACAAATAGGGGTTCCGCGATGCTGAATGAGGGCATCGTTC |
| pET28a-pTA2000-R | GTCTTGAGGGGTTTTTTGTTGCATGGTTTGTTGAAAACCGGAC |
| pET28a-pTA2500-F | ACAAATAGGGGTTCCGCGGCGTTGCCACCTCCAGTC |
| pET28a-pTA2500-R | GGTCTTGAGGGGTTTTTTGCAATCAGCAACGACTGTTTGCC |
| pET28a-pTA3000-F | ACAAATAGGGGTTCCGCGGGCGCCTATATCGCCGAC |
| pET28a-pTA3000-R | GTCTTGAGGGGTTTTTTGAGCAACCGCACCTGTGGC |
| aadA-pTS-F | GCTTCCTTTCGGGCTTTGTTATTTGCCGACTACCTTGGTGATCTC |
| aadA-pTS-R | ATCTCGATCCCGCGAAATTTTGTTTATTTTTCTAAATACATTCAAATATGTATCCGCTC |
| pET28a-pTS-F | ATTTCGCGGGATCGAGATCTCG |
| pET28a-pTS-R | CAAAGCCCGAAAGGAAGCTGAG |
| L33*-F | GGAGCCGTATTAATGTACGGCCAGCAACG |
| L33*-R | CGTACATTaaTACGGCTCCGCAGTGGATG |
| aadA-ORF-F | CAAAAAACCCCTCAAGACCCGTT |
| aadA-ORF-R | TTTGTTTATTTTTCTAAATACATTCAAATATGTATCCGCTCATGAG |
| pET28a-pTS250-F | CATATTTGAATGTATTTAGAAAAATAAACAAAACATGTGAGCAAAAGGCCAGC |
| pET28a-pTS250-R | GGTCTTGAGGGGTTTTTTGTCTTTCCTGCGTTATCCCCTGATTCTG |
| pET28a-pTS1000-F | CATATTTGAATGTATTTAGAAAAATAAACAAAGAAGCGCTGGCATTGACC |
| pET28a-pTS1000-R | TCTTGAGGGGTTTTTTGGTTAATACAGATGTAGGTGTTCCACAGGGTAG |
| Up-arm-A134V-F | aagggcactatgactccaaagaag |
| Up-arm-A134V-R | agaatcctgccaaatggccATcagcaggttgtcctgacgctc |
| Down-arm-A134V-F | ATggccatttggcaggattctaaaggtttcggctacgcgacg |
| Down-arm-A134V-R | gtgccagaatacgttccaggtcg |
| sg-F | TATCTGGCAGGACAGCAAgttttagagctagaaatagcaagttaaaataaggc |
| sg-R | TTGCTGTCCTGCCAGATAGCactagtattatacctaggactgagctagctg |
| lpp-F | gtaaagccgtttttgttgtttcatCGACCCACCGCCTCCG |
| lpp-R | atgggcaaagctactaaactggtactg |
| BLA-F | ctatctttgaacataaattgaaaccgacccg |
| BLA-R | atgaaacaacaaaaacggctttacgc |
| rhaBAD-F | cagtttagtagctttgcccatggtatatctccttcttaaagttaaacaaaGGATCCTG |
| rhaBAD-R | ttaatctttctgcgaattgagatgacgccac |
| pET28a-BLA-F | gtcggtttcaatttatgttcaaagatagCAAAGCCCGAAAGGAAGCTGAG |
| pET28a-BLA-R | gtcatctcaattcgcagaaagattaaGCGCAACGCAATTAATGTAAGTTAGC |
| rhaSR-F | CCGGATATAGTTCCTCCTTTCAGatgtgatgctCACCGCATTTCC |
| rhaSR-R | ttaatctttctgcgaattgagatgacgccac |
| pET28a-rhaSR-F | gtcatctcaattcgcagaaagattaaGCGCAACGCAATTAATGTAAGTTAGC |
| pET28a-rhaSR-R | cacaatttgctgaattgtggtgAACGCCAGCAACGCG |
| BLA-ori-F | caccacaattcagcaaattgtgaacatc |
| BLA-ori-R | CCGGATATAGTTCCTCCTTTCAGC |
| pET28a-ori-F | GCTGAAAGGAGGAACTATATCCGGTTTCCATAGGCTCCGCCCC |
| pET28a-ori-R | CTGAAAGGAGGAACTATATCCGGATTG |
| lpp-ompA-delete-F | gaaggagatataccatgaaacaacaaaaacggctttacgc |
| lpp-ompA-delete-R | gttgtttcatggtatatctccttcttaaagttaaacaaaGGATCCTG |
| BLA-6*His tag-F | agaCATCACCATCATCACCACtagCTGCTGCCACCGCTG |
| BLA-6*His tag-R | ctaGTGGTGATGATGGTGATGtctttgaacataaattgaaaccgacccgc |
| sts-F | cactaacaaatagctggtggaatatATGGCAAGCGTTGAAGAATTTCG |
| sts-R | TTGCCTTCATCAGTTCGTAACCATCGGAATGCTATG |
| 4cl-F | GGTTACGAACTGATGAAGGCAAATGGCGCCGCAGGAAC |
| 4cl-R | TTACAGGCCGTTCGCCAGTTTC |
| P_gap_-F | ttgctcacatctcactttaatcgtgc |
| P_gap_-R | atattccaccagctatttgttagtgaataaaagg |
| pETDuet-F1 | CTGGCGAACGGCCTGTAATTAACCTAGGCTGCTGCCACC |
| pETDuet-R1 | cgattaaagtgagatgtgagcaaATTTCGCGGGATCGAGATCG |
| TtgR-F | GAATTAATCAAAGGTGAGAGGCACatggtgcgtcgcaccaaag |
| TtgR-R | ttatttgcgcagcgccgg |
| PUTR _rpsU_-F | GTGCCTCTCACCTTTGATTAATTCGG |
| PUTR _rpsU_-R | GTGCCTCTCACCTTTGATTAATTCGG |
| pCDFDuet-F1 | ccggcgctgcgcaaataaCTGCTGCCACCGCTGAG |
| pCDFDuet-R1 | CAAAGTTTTACATCAACCCGCATATTTCCTAATGCAGGAGTCGCATAAG |
| P_ttg_-mCherry-F | ttacttgtacagctcgtccatgcc |
| P_ttg_-mCherry-R | accatgaatgtaagtatattccttagcaacatttaactttaagaaggagatatacatatggtgagcaagggcgag |
| pCDFDuet-TtgR-F | gctaaggaatatacttacattcatggttgtttgtaaatactgctgggtgATGCGGGTTGATGTAAAACTTTGTTCG |
| pCDFDuet-TtgR-R | catggacgagctgtacaagtaaATTTCCTAATGCAGGAGTCGCATAAG |
| P_gap_-STS-4CL-F | ttgctcacatctcactttaatcgtgc |
| P_gap_-STS-4CL-R | CCGCGTTGCTGGCGTTCAAAAAACCCCTCAAGACCCG |
| pETDuet-ori-F2 | AACGCCAGCAACGCGG |
| pETDuet-ori-R2 | cgattaaagtgagatgtgagcaaTTTCCATAGGCTCCGCCCC |
| (m) pETDuet-F | gaaaagttcttctcccttacccatatattccaccagctatttgttagtgaataaaagg |
| (m) pETDuet-R | AACGCCAGCAACGCGATC |
| EGFP-pET-F | atgggtaagggagaagaacttttcac |
| EGFP-pET-R | GATCGCGTTGCTGGCGTTTGCTTCTCAAATGCCTGAGGTTTCAG |
| pETTest1-F | CCAAATACTGTTCTTCTAGTGTAGCCGTAGTTAGGCC |
| pETTest1-R | CTACACTAGAAGAACAGTATTTGGTATCTGCGCTCTGC |
| pETTest2-F | ATCGCTGACGTCGGTACCC |
| pETTest2-R | GTACCGACGTCAGCGATCGCGTTGCTGGCGTTCAA |

**Table S4 DNA sequences used in this study**

| Name | Sequence |
| --- | --- |
| cI857 | ATGAGCACAAAAAAGAAACCATTAACACAAGAGCAGCTTGAGGACGCACGTCGCCTTAAAGCAATTTATGAAAAAAAGAAAAATGAACTTGGCTTATCCCAGGAATCTGTCGCAGACAAGATGGGGATGGGGCAGTCAGGCGTTGGTGCTTTATTTAATGGCATCAATGCATTAAATGCTTATAACGCCGCATTGCTTACAAAAATTCTCAAAGTTAGCGTTGAAGAATTTAGCCCTTCAATCGCCAGAGAAATCTACGAGATGTATGAAGCGGTTAGTATGCAGCCGTCACTTAGAAGTGAGTATGAGTACCCTGTTTTTTCTCATGTTCAGGCAGGGATGTTCTCACCTAAGCTTAGAACCTTTACCAAAGGTGATGCGGAGAGATGGGTAAGCACAACCAAAAAAGCCAGTGATTCTGCATTCTGGCTTGAGGTTGAAGGTAATTCCATGACCGCACCAACAGGCTCCAAGCCAAGCTTTCCTGACGGAATGTTAATTCTCGTTGACCCTGAGCAGGCTGTTGAGCCAGGTGATTTCTGCATAGCCAGACTTGGGGGTGATGAGTTTACCTTCAAGAAACTGATCAGGGATAGCGGTCAGGTGTTTTTACAACCACTAAACCCACAGTACCCAATGATCCCATGCAATGAGAGTTGTTCCGTTGTGGGGAAAGTTATCGCTAGTCAGTGGCCTGAAGAGACGTTTGGCTGA |
| cI857* | ATGAGCACAAAAAAGAAACCATTAACACAAGAGCAGCTTGAGGACGCACGTCGCCTAAAGCAATTTATGAAAAAAAGAAAAATGAACTTGGCTTATCCCAGGAATCTGTCGCAGACAAGATGGGGATGGGGCAGTCAGGCGTTGGTGCTTTATTTAATGGCATCAATGCATTAAATGCTTATAACGCCGCATTGCTTACAAAAATTCTCAAAGTTAGCGTTGAAGAATTTAGCCCTTCAATCGCCAGAGAAATCTACGAGATGTATGAAGCGGTTAGTATGCAGCCGTCACTTAGAAGTGAGTATGAGTACCCTGTTTTTTCTCATGTTCAGGCAGGGATGTTCTCACCTAAGCTTAGAACCTTTACCAAAGGTGATGCGGAGAGATGGGTAAGCACATCCAAAAAAGCCAGTGATACTGCATTCTGGCTTGAGGTTGAAGGTAATTCCATGACCGCACCAACAGGCTCCAAGCCAAGCTTTCCTGACGGAATGTTAATTCTCGTTGACCCTGAGCAGGCTGTTGAGCCAGGTGATTTCTGCATAGCCAGACTTGGGGGTGATGAGTTTACCTTCAAGAAACTGATCAGGGATAGCGGTCAGGTGTTTTTACAACCACTAAACCCACAGTACCCAATGATCCCATGCAATGAGAGTTGTTCCGTTGTGGGGAAAGTTATCGCTAGTCAGTGGCCTGAAGAGACGTTTGGCTGA |
| Pol I | atggttcagatcccccaaaatccacttatccttgtagatggttcatcttatctttatcgcgcatatcacgcgtttcccccgctgactaacagcgcaggcgagccgaccggtgcgatgtatggtgtcctcaacatgctgcgcagtctgatcatgcaatataaaccgacgcatgcagcggtggtctttgacgccaagggaaaaacctttcgtgatgaactgtttgaacattacaaatcacatcgcccgccaatgccggacgatctgcgtgcacaaatcgaacccttgcacgcgatggttaaagcgatgggactgccgctgctggcggtttctggcgtagaagcggacgacgttatcggtactctggcgcgcgaagccgaaaaagccgggcgtccggtgctgatcagcactggcgataaagatatggcgcagctggtgacgccaaatattacacttatcaataccatgacgaataccatcctcggaccggaagaggtggtgaataagtacggcgtgccgccagaactgatcatcgatttcctggcgctgatgggtgactcctctgataacattcctggcgtaccgggcgtcggtgaaaaaaccgcgcaggcattgctgcaaggtcttggcggactggatacgctgtatgccgagccagaaaaaattgctgggttgagcttccgtggcgcgaaaacaatggcagcgaagctcgagcaaaacaaagaagttgcttatctctcataccagctggcgacgattaaaaccgacgttgaactggagctgacctgtgaacaactggaagtgcagcaaccggcagcggaagagttgttggggctgttcaaaaagtatgagttcaaacgctggactgctgatgtcgaagcgggcaaatggttacaggccaaaggggcaaaaccagccgcgaagccacaggaaaccagtgttgcagacgaagcaccagaagtgacggcaacggtgatttcttatgacaactacgtcaccatccttgatgaagaaacactgaaagcgtggattgcgaagctggaaaaagcgccggtatttgcatttgataccgaaaccgacagccttgataacatctctgctaacctggtcgggctttcttttgctatcgagccaggcgtagcggcatatattccggttgctcatgattatcttgatgcgcccgatcaaatctctcgcgagcgtgcactcgagttgctaaaaccgctgctggaagatgaaaaggcgctgaaggtcgggcaaaacctgaaatacgCtcgcggtattctggcgaactacggcattgaactgcgtgggattgcgtttgataccatgctggagtcctacattctcaatagcgttgccgggcgtcacgatatggacagcctcgcggaacgttggttgaagcacaaaaccatcacttttgaagagattgctggtaaaggcaaaaatcaactgacctttaaccagattgccctcgaagaagccggacgttacgccgccgaagatgcagatgtcaccttgcagttgcatctgaaaatgtggccggatctgcaaaaacacaaagggccgttgaacgtcttcgagaatatcgaaatgccgctggtgccggtgctttcacgcattgaacgtaacggtgtgaagatcgatccgaaagtgctgcacaatcattctgaagagctcacccttcgtctggctgagctggaaaagaaagcgcatgaaattgcaggtgaggaatttaacctttcttccaccaagcagttacaaaccattctctttgaaaaacagggcattaaaccgctgaagaaaacgccgggtggcgcgccgtcaacgtcggaagaggtactggaagaactggcgctggactatccgttgccaaaagtgattctggagtatcgtggtctggcgaagctgaaatcgacctacaccgacaagctgccgctgatgatcaacccgaaaaccgggcgtgtgcatacctcttatcaccaggcagtaactgcaacgggacgtttatcgtcaaccgatcctaacctgcaaaacattccggtgcgtaacgaagaaggtcgtcgtatccgccaggcgtttattgcgccagaggattatgtgattgtctcagcggactactcgcagattgaactgcgcattatggcgcatctttcgcgtgacaaaggcttgctgaccgcattcgcggaaggaaaagatatccaccgggcaacggcggcagaagtgtttggtttgccactggaaaccgtcaccagcgagcaacgccgtagcgcgaaagcgatcaactttggtctgatttatggcatgagtgctttcggtctggcgcggcaattgaacattccacgtaaagaagcgcagaagtacatggacctttacttcgaacgctaccctggcgtgctggagtatatggaacgcacccgtgctcaggcgaaagagcagggctacgttgaaacgctggacggacgccgtctgtatctgccggatatcaaatccagcaatggtgctcgtcgtgcagcggctgaacgtgcagccattaacgcgccaatgcagggaaccgccgccgacattatcaaacgggcgatgattgccgttgatgcgtggttacaggctgagcaaccgcgtgtacgtatgatcatgcaggtacacgatgaactggtatttgaagttcataaagatgatgttgatgccgtcgcgaagcagattcatcaactgatggaaaactgtacccgtctggatgtgccgttgctggtggaagtggggagtggcgaaaactgggatcaggcgcactaa |
| BLA | atgaaacaacaaaaacggctttacgcccgattgctgacgctgttatttgcgctcatcttcttgctgcctcattctgcagcagcggcggcaaatcttaatgggacgctgatgcagtattttgaatggtacacgcccaatgacggccaacattggaagcgtttgcaaaacgactcggcatatttggctgaacacggtattactgccgtctggattcccccggcatataagggaacgagccaagcggatgtgggctacggtgcttacgacctttatgatttaggggagtttcatcaaaaagggacggttcggacaaagtacggcacaaaaggagagctgcaatctgcgatcaaaagtcttcattcccgcgacattaacgtttacggggatgtggtcatcaaccacaaaggcggcgctgatgcgaccgaagatgtaaccgcggttgaagtcgatcccgctgaccgcaaccgcgtaatttccggagaatacctaattaaagcctggacacattttcattttccggggcgcggcagcacatacagcgattttaaatggcattggtaccattttgacggaaccgattgggacgagtcccgaaagctgaaccgcatctataagtttcaaggaaaggcttgggattgggaagtttccagtgaaaacggcaactatgattatttgatgtatgccgacatcgattatgaccatcctgatgtcgtagcagaaattaagagatggggcacttggtatgccaatgagctccaattggacggtttccgtcttgatgctgtcaaacacattaaattttcttttttgcgggattgggttaatcatgtcagggaaaaaacggggaaggaaatgtttacggtagctgaatattggcagaatgacttgggcgcgctggaaaactatttgaacaaaacaaattttaatcattcagtgtttgacgtgccgcttcattatcagttccatgctgcatcgacacagggaggcggctatgatatgaggaaattgctgaacggtacggtcgtttccaagcatccgttgaaatcggttacatttgtcgataaccatgatacacagccggggcagtcgcttgagtcgactgtccaaacatggtttaagccgcttgcttacgcttttattctcacaagggaatctggataccctcaggttttctacggggatatgtacgggacgaaaggagactcccagcgcgaaattcctgccttgaaacacaaaattgaaccgatcttaaaagcgagaaaacagtatgcgtacggagcacagcatgattatttcgaccaccatgacattgtcggctggacaagggaaggcgacagctcggttgcaaattcaggtttggcggcattaataacagacggacccggtggggcaaagcgaatgtatgtcggccggcaaaacgccggtgagacatggcatgacattaccggaaaccgttcggagccggttgtcatcaattcggaaggctggggagagtttcacgtaaacggcgggtcggtttcaatttatgttcaaagatag |
| STS | ATGGCAAGCGTTGAAGAATTTCGTAATGCTCAGCGTGCAAAAGGCCCGGCGACCATCCTGGCGATTGGCACGGCTACCCCGGACCACTGTGTGTATCAGAGCGATTACGCCGACTTTTATTTCCGTGTTACCAAATCTGAACACATGACGGCACTGAAAAAGAAATTCAACCGTATTTGCGATAAGTCAATGATTAAGAAACGCTACATCCATCTGACCGAAGAAATGCTGGAAGAACACCCGAACATTGGCGCTTATATGGCGCCGTCGCTGAATATCCGCCAGGAAATTATCACGGCTGAAGTGCCGAAACTGGGCAAGGAAGCGGCCCTGAAAGCGCTGAAGGAATGGGGTCAACCGAAATCAAAGATTACCCATCTGGTCTTCTGTACCACGTCGGGCGTGGAAATGCCGGGTGCAGATTACAAACTGGCAAATCTGCTGGGTCTGGAACCGAGCGTGCGTCGCGTTATGCTGTACCACCAGGGTTGCTATGCCGGCGGTACCGTTCTGCGTACGGCTAAAGATCTGGCGGAAAACAATGCAGGCGCTCGCGTCCTGgTGGTTTGTAGCGAAATTACCGTCGTGACGTTTCGTGGCCCGAGCGAAGATGCCCTGGACTCTCTGGTGGGTCAAGCACTGTTCGGCGATGGTTCTGCAGCTGTTATCGTCGGTTCAGATCCGGACATTTCGATCGAACGTCCGCTGTTTCAGCTGGTTAGCGCGGCCCAAACCTTCATTCCGAACTCCGCAGGTGCAATCGCAGGTAATCTGCGCGAAGTTGGCCTGACCTTTCACCTGTGGCCGAACGTCCCGACGCTGATTAGCGAAAATATCGAAAAATGCCTGACCCAGGCATTTGATCCGCTGGGTATTAGTGACTGGAACTCCCTGTTCTGGATTGCTCATCCGGGCGGTCCGGCAATCCTGGATGCAGTGGAAGCAAAACTGAACCTGGACAAGAAAAAGCTGGAAGCGACCCGCCACGTCCTGAGTGAATATGGCAATATGAGCTCTGCCTGTGTGCTGTTTATCCTGGATGAAATGCGTAAAAAGTCCCTGAAAGGTGAACGTGCAACCACGGGTGAAGGTCTGGACTGGGGCGTTCTGTTTGGTTTCGGTCCGGGTCTGACGATTGAAACGGTGGTCCTGCATAGCATTCCGATGGTTACGAACTGA |
| 4CL | ATGGCGCCGCAGGAACAGGCGGTTTCTCAGGTTATGGAAAAACAGTCTAACAACAACAACAGCGACGTTATCTTCCGTAGCAAACTGCCGGATATTTACATCCCGAACCATCTGTCTCTGCACGATTACATCTTTCAGAACATTTCTGAATTTGCGACTAAACCGTGCCTGATTAACGGCCCGACCGGTCACGTGTACACCTACAGCGATGTTCACGTGATCAGCCGCCAGATTGCGGCCAACTTCCACAAACTGGGCGTGAACCAGAACGATGTTGTGATGCTGCTGCTGCCGAACTGCCCGGAATTTGTTCTGTCTTTCCTGGCGGCAAGCTTTCGTGGTGCTACCGCAACCGCGGCTAACCCGTTCTTCACCCCGGCGGAAATCGCTAAACAGGCAAAAGCTTCTAACACTAAACTGATCATCACTGAAGCGCGCTATGTTGATAAAATCAAACCGCTGCAGAACGATGACGGTGTTGTTATTGTGTGCATCGATGATAACGAATCCGTGCCAATCCCGGAAGGCTGCCTGCGTTTCACCGAACTGACCCAGTCCACCACCGAAGCGTCCGAAGTGATTGATAGCGTGGAAATTTCTCCGGATGATGTAGTGGCGCTGCCGTACTCTAGCGGTACTACCGGTCTGCCGAAAGGTGTTATGCTGACCCATAAAGGTCTGGTTACTTCCGTTGCACAGCAGGTTGATGGTGAAAACCCGAACCTGTACTTCCACTCTGATGATGTGATCCTGTGCGTTCTGCCGATGTTCCACATCTACGCACTGAACTCTATTATGCTGTGTGGCCTGCGTGTAGGTGCTGCAATTTTGATCATGCCGAAATTCGAAATCAACCTGTTGCTGGAACTGATCCAGCGTTGCAAAGTGACCGTTGCGCCGATGGTTCCGCCGATCGTTCTGGCAATTGCTAAAAGCTCTGAAACCGAAAAATACGACCTGAGCTCTATCCGCGTTGTTAAAAGCGGTGCGGCACCGCTGGGTAAAGAACTGGAAGATGCTGTTAACGCTAAATTCCCGAACGCGAAACTGGGCCAGGGTTACGGCATGACCGAAGCTGGTCCGGTGCTGGCGATGAGCCTGGGCTTCGCTAAAGAACCGTTTCCGGTTAAATCCGGCGCATGTGGTACCGTGGTTCGTAACGCTGAAATGAAAATCGTTGATCCGGATACCGGTGATAGCCTGTCTCGTAACCAGCCGGGTGAAATTTGCATTCGTGGTCATCAGATCATGAAAGGCTATCTGAACAATCCGGCGGCGACCGCGGAAACTATCGATAAAGATGGCTGGCTGCACACCGGTGACATCGGTCTGATCGATGACGATGATGAACTGTTTATCGTAGACCGTCTGAAAGAACTGATTAAATACAAAGGCTTCCAGGTGGCGCCGGCTGAACTGGAAGCTCTGCTGATCGGCCACCCGGATATCACCGATGTTGCGGTGGTTGCGATGAAAGAAGAAGCGGCGGGTGAAGTTCCGGTTGCGTTCGTTGTTAAATCTAAAGACTCTGAACTGAGCGAAGATGATGTTAAACAGTTCGTGAGCAAACAGGTTGTGTTTTATAAACGTATTAACAAAGTTTTCTTCACCGAAAGCATCCCGAAAGCGCCGTCTGGCAAAATCCTGCGTAAAGACCTGCGTGCGAAACTGGCGAACGGCCTGTAA |
| ttgR | ATGGTGCGTCGCACCAAAGAAGAAGCACAGGAAACGCGTGCGCAGATTATCGAAGCGGCCGAACGCGCGTTTTATAAACGTGGTGTGGCACGTACCACGCTGGCAGATATTGCAGAACTGGCAGGTGTTACCCGCGGTGCAATCTACTGGCATTTCAACAATAAAGCCGAACTGGTTCAGGCACTGCTGGATTCTCTGCACGAAACGCATGATCACCTGGCCCGTGCAAGCGAATCTGAAGATGAACTGGACCCGCTGGGCTGCATGCGCAAACTGCTGCTGCAGGTGTTTAACGAACTGGTTCTGGATGCACGTACCCGTCGCATTAATGAAATCCTGCATCACAAATGCGAATTTACGGATGATATGTGTGAAATTCGTCAGCAGCGCCAGAGCGCCGTGCTGGATTGTCATAAAGGTATCACCCTGGCACTGGCAAACGCAGTTCGTCGCGGTCAGCTGCCGGGTGAACTGGATGTGGAACGCGCAGCGGTTGCGATGTTTGCCTATGTGGATGGCCTGATTGGTCGTTGGCTGCTGCTGCCGGATAGTGTTGACCTGCTGGGCGATGTGGAAAAATGGGTTGATACCGGTCTGGATATGCTGCGTCTGAGCCCGGCGCTGCGCAAATAA |
